# Innovative manure via hyper-thermophilic fermentation coupled with heat-resistant phosphate-solubilizing Bacillus inoculation promotes phosphorus transformation by assembling keystone taxa in the oat rhizosphere

**DOI:** 10.1128/aem.01208-25

**Published:** 2025-12-29

**Authors:** Chengzhen Zhao, Xiao Chang, Lili Fan, Linshu Jiang, Rongzhen Zhong

**Affiliations:** 1 Jilin Province Feed Processing and Ruminant Precision Breeding Cross regional Cooperation Technology Innovation Center, Jilin Provincial Laboratory of Grassland Farming, Northeast Institute of Geography and Agroecology, Chinese Academy of Sciences 66276 , Changchun, China; 2 University of Chinese Academy of Sciences 12381, Beijing, China; Kyoto University, Kyoto, Japan

**Keywords:** organic manures, phosphate-solubilizing microorganism, hyper-thermophilic fermentation, phosphorus transformation, Bacillus, Thermobifida

## Abstract

Phosphorus forms and distribution in organic manures vary under different treatment conditions, thereby exerting distinct effects on the soil microbiome and soil phosphorus transformation process. This study examined the effects of a novel manure treated with hyper-thermophilic fermentation combined with Bacillus strain inoculation, compared with raw and composted manure, on the oat rhizosphere microbiome and phosphorus transformation across different soil types in a controlled pot experiment. Our findings demonstrate that hyper-thermophilic fermentation with Bacillus inoculation not only promotes the survival and abundance of the bacterial genus Bacillus but also selectively enriches the hyper-thermophilic bacterial genus Thermobifida in the fermented manure. Notably, the application of hyper-thermophilic fermented manure led to a significant enrichment of keystone species like Bacillus and Thermobifida across both soil types, relative to other manure applications. These genera emerged as key drivers of available phosphorus, phosphatase activity, and differential metabolites in the rhizosphere, exhibiting a synergistic effect on soil phosphorus transformation. Fermented manure exhibited superior performance relative to conventional composted manure, as it increased the phosphorus uptake rate of oats by 35.5% in black soil and 27.9% meadow soil, respectively, over a single growing season. Additionally, among all organic manures, the application of fermented manure significantly enhanced the sequestration of phosphorus from manure in the soils, with 78.0% in black soil and 56.9% in meadow soil. This consequently reduced *P* loss to 13.6% and 34.4% in the respective soil types.

**IMPORTANCE:**

Phosphate-solubilizing microorganisms (PSMs) are frequently proposed as catalysts for promoting phosphorus recycling; however, their performance is often inefficient or ineffective in the context of a circular bioeconomy within agricultural systems. This study introduces innovative concepts and methodologies by integrating hyper-thermophilic fermentation with heat-resistant phosphate-solubilizing Bacillus inoculation, thereby enhancing the effective phosphorus recovery and utilization from manure waste in sustainable agricultural practices.

## INTRODUCTION

Food security—defined by the availability of food and individuals’ ability to access it—influences the sustainable development, future, and destiny of mankind. The rapid growth of the human population dictates a rising demand for food (1). However, the global decline in soil fertility has resulted in decreased crop yields, which poses a permanent challenge for humankind. In the past decades, although increasing doses of chemical fertilizers have improved agricultural productivity, especially in the short term, they often lead to soil compaction and acidification, endanger farm-related biodiversity, decrease soil quality, and pose health risks to consumers (2, 3). Modern agriculture aims to enhance soil quality and sustainable productivity with a focus on organic manure (4).

The application of organic manures has become a mainstream method in modern agriculture due to its advantages in implementing material recycling, avoiding the accumulation and dissipation of fertilizing nutrients in the environment, and maintaining soil fertility. This approach is recommended not only by the European Union but also by the European Industrial Organization of Fertilizers (5). However, organic manures are not always as productive in terms of yields as conventional chemical fertilizers, thus offsetting their advantages to some extent. Both enlarging cultivable areas and increasing doses of organic manure have their limits, especially under the extreme constraints of arable land availability (6). Therefore, an important strategy in modern agriculture is to increase the nutrient use efficiency of organic manure. The most critical aspect of this strategy is to enhance their nutrient availability, especially for phosphorus. Phosphorus is an essential mineral nutrient for plant growth, possessing significant nutritional and physiological functions. The main form of available phosphorus, named orthophosphate, is easily absorbed and utilized by plants (7). In most soils, phosphorus availability is low and limits crop yield, as it is readily inactivated through precipitation/adsorption, storage, and biological fixation with clay particles and metal ions, such as aluminum-P, iron-P, and residual phosphorus (8).

Organic manures are an important external source of soil phosphorus and serve as an organic amendment when incorporated into soils (9, 10). The majority of studies have reported that the application of organic manures increases phosphorus transformation, making phosphorus more available in agricultural soils (11, 12). In general, organic manure fertilization can increase phosphorus availability in soils by directly releasing its own available phosphorus (13) and by enhancing soil microbial activity, which promotes the transformation of insoluble inorganic phosphorus and organic phosphorus (14). Organic manures prepared by different methods differ in their own phosphorus availability as well as in the biological transformation of phosphorus in the plant rhizosphere. Direct incorporation of raw organic manure into soils is a convenient way to achieve the recycling of phosphorus in the soil-plant system, but it has low phosphorus availability for plants, as most of the applied phosphorus from raw manure is quickly fixed by soil microorganisms and ultimately lost to the environment (5). Composting is a widely used technique to increase microbial aerobic activity and prepare P-enriched compost products, which enhances and improves the soil structure, increases crop phosphorus uptake, and increases soil phosphorus availability (15, 16). Compared with the slow heating rate of traditional composting, hyper-thermophilic fermentation with the inoculation of hyper-thermophilic microorganisms has been suggested as a potential innovative approach for accelerating the composting process (17). More importantly, the plant rhizosphere is a hotspot of microbial activity, with a highly active flow of nutrients and information due to frequent plant-microbe interactions (18). Rhizosphere microorganisms are essential biological factors that promote phosphorus transformation under organic manure fertilization, thus determining the phosphorus utilization efficiency and supporting sustainable agriculture (19).

Rhizosphere microorganisms, especially phosphate-solubilizing microorganisms (PSMs), can utilize phosphorylated compounds as a carbon source and thus mineralize organic phosphorus through the production and activities of extracellular enzymes such as acid and alkaline phosphatase and phytase (20, 21). PSMs can also secrete significant amounts of organic acids, which are the primary low-molecular-weight carbon-containing metabolites that promote the transformation of insoluble phosphorus (22). Although PSMs have the potential capacity to mineralize organic phosphorus and solubilize insoluble phosphorus, this process is difficult to regulate. Some studies have attempted to inoculate PSMs into agricultural soils to promote phosphorus transformation efficiency and thus improve crop yield; however, the results are inconsistent. On one hand, some studies showed that inoculation with PSMs could increase plant access to phosphorus from organic fertilizers (23). On the other hand, some studies showed no effect, and in some cases, negative effects on inoculation with PSMs, due largely to poor colonization (24). This indicates that there is a high level of complexity and intense competition among microorganisms in the rhizosphere environment (25). A previous study reported that a manure composting pile was an excellent habitat for microbial growth and reproduction and that high temperatures could specifically favor the survival of hyper-thermophilic microorganisms and reduce competition from other microorganisms (26). Based on this, it remains to be assessed whether innovatively inoculating heat-resistant, domesticated PSM strains into manure waste during hyper-thermophilic fermentation could strengthen PSM accumulations and promote phosphorus speciation transformation in organic manure. Moreover, the actual impact of this fermented organic manure, compared with raw and composted manure, on rhizosphere phosphorus transformation and microbiome composition remains unknown. Additionally, rhizosphere microorganisms and their abundance vary significantly across soil types, which may also exert a substantial effect on phosphorus transformation under organic manure incorporation. Therefore, this study aims to further investigate the impact of different organic manures on the rhizosphere microbiome in various soils, thereby providing a general application strategy for agricultural soils.

In the current study, we use oats (Avena sativa “Baiyan No. 2”) grown in black and meadow soils in pots as a model to evaluate organic manure application in agricultural settings. The objectives of this study are to (i) determine the phosphorus transfer from organic manure to oats and soils under different organic manure fertilization regimes; (ii) investigate the changes in rhizosphere microbiome, phosphatase activity, and rhizosphere metabolites following the incorporation of organic manures; and (iii) identify the keystone microorganisms in rhizosphere microbial communities that drive soil phosphorus transformation after incorporating organic manure.

## MATERIALS AND METHODS

### Organic manure preparation methods

#### Feedstock

Fresh cattle manure was obtained from Ling Yan Breeding Farm, Gongzhuling, Jilin, China (44°34'N, 123°35'E) and was immediately stored at −20℃. Corn straw, harvested at the ripening stage from a local field, was initially cut into lengths of 2–5 cm and subsequently crushed.

Prior to the preparation of three organic manures, the composition of each feedstock material pile adhered to a recommended formula (27), comprising manure (3.2 kg DM), straw (9.6 kg DM), urea (0.15 kg), lime (0.08 kg), and gypsum (0.08 kg). Each pile was thoroughly mixed to achieve a homogenized feedstock mixture with an adjusted moisture content of approximately 63%.

#### Raw manure preparation methods

Fresh manure, without any treatment, served as the control and is termed raw manure.

#### Composted manure preparation methods

The composting process and methods employed are detailed in our previous study (28). Briefly, the feedstock mixture pile was placed in a high-density polyethylene bucket (40 cm diameter, 80 cm high, ~100 L) without a lid. Six replicates were used for the aerobic composting treatment. All buckets were stored in a greenhouse for 56 days. This location was naturally ventilated and maintained temperature and humidity within a range of 25.0°C–30.0°C and 60%–70%, respectively. During the composting period, the compost pile in each bucket was turned once every 3 days during the first week and subsequently once a week until the end, as specified in (27). The composted manure was obtained following the completion of the conventional aerobic composting process described above.

#### Hyper-thermophilic fermented manure preparation methods

Before fermentation, each feedstock mixture pile was inoculated with ultra-temperature-resistant phosphate-solubilizing microorganisms (PSMs), including strains of Bacillus subtilis, Bacillus licheniformis, and Bacillus megaterium. These PSM strains were previously screened and adapted using a temperature gradient method to enhance their activity at temperatures of 85°C–95°C, demonstrating stability in ultra-temperature resistance and phosphatase properties across several generations. The mixed PSMs agent was inoculated at a rate of 0.3% based on the dry weight of the feedstock mixture at the outset of fermentation (29). The feedstock mixture pile (12.8 kg DM) and mixed PSM inoculant (38.4 g DM) were thoroughly combined. The bacterial concentration of the PSMs agent was 1 × 10^7^ CFU/g, consisting of *Bacillus subtilis*, *Bacillus licheniformis*, and *Bacillus megaterium* in a 1:1:1 mass ratio. The fermented systems and operational methods used are described in our previously authorized patent (30, 31). In summary, the feedstock mixture pile was placed in a small fermentation tank (40 cm diameter, 80 cm high, ~100 L) within an automated fermentation system. Six replicates were used in the hyper-thermophilic fermentation treatment. The automated fermentation process involved a heating stage lasting 2–4 h to raise the temperature to 85°C–95°C, followed by a maintenance phase of 24–36 h at this temperature range, and concluding with a cooling phase of 6–12 h to ambient temperature. A circulating hot air return device facilitated temperature regulation and oxygen circulation, whereas the main shaft rotated continuously to ensure thorough mixing of materials and oxygen. The fermented manure was obtained following completion of the innovative hyper-thermophilic fermentation process described above.

#### Organic manure sample collection and analysis

For each type of organic manure, six replicated subsamples were collected, with each subsample divided into four portions. One portion was dried at 65°C for 72 h and ground to pass through a 40-mesh sieve for phosphorus fraction determination. One portion was stored at 4°C for the analysis of microbial biomass phosphorus concentration. Another was stored at −20°C for phosphatase activity analysis, and the final portion was stored at −80°C for microbial analysis. The analytical methods were consistent with those described for soil samples.

### Controlled pot experiment

#### Raw soil collection and analysis

Two raw soil collection sites were selected to represent the main agricultural production areas in the eastern and western parts of Northeast China (Fig. S4). These sites included black soil in Changchun (Jilin Province, 43°59′N, 125°23′E, a typical black soil area) and meadow soil in Changling (Jilin Province, 44°33′N, 123°31′E, a typical agro-pastoral transition area). The average annual temperature and precipitation in Changchun are 6.4℃ and 614 mm, respectively, compared with 5.9℃ and 427 mm in Changling. Raw black and meadow soils from the top 25 cm were collected from maize crop farmland before planting on June 20, 2022. Only maize is planted in these sites during a single growing season each year due to the cold climate. The black soil is classified as clay loam, containing approximately 36.0% clay, 24.5% silt, and 39.5% sand (Typic Hapludoll, USDA Soil Taxonomy). The meadow soil is an alkali-saline soil with a texture comprising 42% clay, 35% silt, and 23% sand (World Reference Base for Soil Resources). The basic physicochemical characteristics of the raw black soil and meadow soil are presented in Fig. 2. The collected soils were sieved through a 2 mm mesh, thoroughly mixed, and stored under natural conditions in a greenhouse prior to their use in a pot experiment.

#### Pot experiment site, design, and management

The pot experiment was conducted in an automated greenhouse at the Agricultural Ecology Station of the Northeast Institute of Geography and Agroecology, Chinese Academy of Sciences, located in Changchun City, Jilin Province, China (43°59′N, 125°23′E). The experiment involved four fertilization treatments: no manure addition (CK), composted manure addition (CM), fermented manure addition (FM), and raw manure addition (RM). Each treatment was replicated six times, using pots measuring 20 cm in diameter and 25 cm in height, across two soil types—black soil and meadow soil. This setup resulted in a total of eight treatments across 48 pots. The CM fertilizer was derived from fresh cattle manure that had been composted for 8 weeks. In contrast, the FM fertilizer originated from fresh cattle manure subjected to ultra-thermophilic fermentation for 48 h. Untreated fresh cattle manure was used as RM fertilizer. Further details on the preparation methods of these three types of organic fertilizers are available in the Supplementary Materials and Methods. Each type of soil, black and meadow, was packed into 24 pots, making a total of 48 pots. To control for moisture content across the three fertilizers and two soil types, they were added based on their actual dry matter weights. Each pot contained 4.2 kg of soil (dry matter). Pots receiving fertilization treatments included an additional 200 g of fertilizer (dry matter), which was thoroughly mixed with the soil. The specific additions of raw soil and fertilizer for each treatment are detailed in Table S2. On July 24, 2022, each pot was sown with 20 oat seeds, which had a germination rate of 90%. After one week, the seedlings were thinned to retain only ten per pot. Throughout the plant growth period, the cultivation was managed under natural conditions supplemented with artificial irrigation, providing equal volumes of water to all pots three times per week.

#### Sample collection and analysis

At the oat heading stage, 12 weeks after planting (October 16, 2022), samples from aboveground biomass, belowground biomass, and rhizosphere soil were collected from all 48 experimental pots. The specific sampling procedures for each pot were as follows. First, all aboveground shoots in each pot were clipped, dried at 65°C for 72 h, weighed to determine aboveground biomass (AB), then sieved through a 40-mesh screen and stored at 4°C for P concentration analysis. Second, the complete root system of the oats was carefully separated from the soil clump. Loosely adhering soil was gently shaken off, and the rhizosphere soil was then collected by brushing off the remaining soil, following the method described by (32). The collected soil was sieved through a 2 mm screen, divided into two parts: one for analyzing soil physicochemical properties and the other stored at −80°C for soil DNA extraction and metabolomic analyses. Finally, the remaining oat root system was dried at 65°C for 72 h, weighed as belowground biomass (BB), then sieved through a 40-mesh screen and stored at 4°C for P concentration analysis.

Soil pH was measured using a pH meter after shaking a 1:2.5 (wt/vol) soil-to-0.01 M CaCl_2_ solution mixture for 30 min. Total phosphorus (TP) and available phosphorus (AP) were determined via HClO_4_-H_2_SO_4_ digestion and NaHCO_3_ extraction methods, respectively, as described by (33) and (34). The concentration of TP and AP contents was subsequently quantified using a continuous flow analytical system (SKALAR San ++, Skalar, Holland). Soil microbial biomass phosphorus (MBP) was analyzed using the fumigation-extraction method (35). For plant samples, the phosphorus concentration in both above-ground and below-ground oat tissues was determined using the molybdate blue colorimetric method after H_2_SO_4_-HClO_4_ digestion, according to (36).

Oat phosphorus uptake (%), soil phosphorus retention (%), and phosphorus loss (%) for three fertilization treatments in both black and meadow soil were calculated according to the method described by (37). The calculations are as follows: above-ground phosphorus accumulation (APA, mg/pot) and below-ground phosphorus accumulation (BPA, mg/pot) in each pot were determined by multiplying the above-ground biomass by the above-ground phosphorus content and the below-ground biomass by the below-ground phosphorus content, respectively. Oat phosphorus uptake (%) from fertilizer was calculated as ([APA + BPA in fertilized pots] − [APA + BPA in control pots])/amount of phosphorus in fertilizer × 100%. Soil phosphorus retention from fertilizer (%) was calculated as ([TP in fertilized pots] – [TP in control pots])/amount of phosphorus in fertilizer × 100%. Phosphorus loss from fertilizer (%) was calculated as 100% − (oat phosphorus uptake [%] + soil phosphorus retention [%]). The amount of phosphorus applied in three fertilization treatments was determined by multiplying the weight of the fertilizer by its phosphorus content.

To facilitate the determination, acid phosphatase (EC 3.1.3.2, ACP) was quantified using an acetate buffer (pH = 5.0 ~ 5.4), neutral phosphatase (NEP) with a citrate buffer (pH = 7.0), and alkaline phosphatase (EC3.1.3.1, ALP) with a borate buffer (pH = 9 ~ 10). The quantification was performed by measuring the amount of *p*-nitrophenol released from samples after incubation at 37°C for 1 h, using *p*-nitrophenyl phosphate as a substrate (38). Phytate activity was determined by measuring the amount of inorganic P liberated from sodium phytate solution, also using sodium phytate as the substrate (39). Phosphatase activities (ACP, NEP, and ALP) were expressed as mg PNP/g, 24 h) dry soil, whereas phytate activity was expressed as μg PNP/g, 24 h) dry soil.

#### Soil DNA extraction, PCR assays, and high-throughput sequencing

Total DNA was extracted from 0.5 g of rhizosphere soil using the OMEGA Soil DNA Kit (M5635-02) (Omega Bio-Tek, Norcross, GA, USA), as per the manufacturer’s instructions, and stored at −20°C prior to further analysis. The quantity and quality of DNA were assessed using a NanoDrop NC2000 spectrophotometer (Thermo Fisher Scientific, Waltham, MA, USA) and agarose gel electrophoresis, respectively. Two commonly used primer sets were applied for metabarcoding approaches to study microbial communities, targeting bacterial 16S rRNA genes and ITS rRNA gene sequences, respectively (Table S2).

PCR reactions were conducted in four parallel reactions in 24 µL mixtures consisting of 5 µL of buffer, 0.25 µL of Fast pfu DNA Polymerase (5 U/µL), 2 µL (2.5 mM) of dNTPs, 1 µL (10 uM) of each Forward and Reverse primer, 1 µL of DNA Template, and 14.75 µL of sterilized ddH_2_O. Negative control samples were also included throughout the PCR assay to ensure reaction systems were not contaminated. PCR conditions for each primer set are detailed in Table S3. PCR amplicons were purified with Vazyme VAHTS DNA Clean Beads (Vazyme, Nanjing, China) and quantified using the Quant-iT PicoGreen dsDNA Assay Kit (Invitrogen, Carlsbad, CA, USA). Purified amplicons were pooled in equimolar amounts and sent for paired-end 2 × 250 bp sequencing on the NovaSeq platform with a NovaSeq 6000 SP Reagent Kit (500 cycles) (Shanghai Personal Biotechnology Co., Ltd., Shanghai, China).

Microbiome bioinformatics were conducted using Quantitative Insights into Microbial Ecology (QIIME) 2.0 (40) with modifications according to the official tutorials (https://docs.qiime2.org/2019.4/tutorials/tutorials/). Specifically, raw sequence data were demultiplexed using the demux plugin, followed by primer trimming with a cutadapt plugin (41). Subsequently, sequences underwent quality filtering, denoising, merging, and chimera removal using the DADA2 plugin (42). The processed sequences were then used to generate amplicon sequence variants (ASVs), with each ASV sequence representing a unique taxonomic assignment. Bacterial and fungal ASVs were classified against the SILVA 132 and UNITE 9.0 databases, respectively, at a minimum similarity threshold of 90%. The evaluation of sample sequencing data for bacteria (Table S4) and fungi (Table S5), along with rarefaction curves for bacterial Fig. S5A and B) and fungal communities (Table S5C and D) confirm that our sequencing data accurately reflects their compositions. Sequences were rarefied to a minimum number of sequences per sample (bacteria: 63,608; fungi: 51,005) for downstream analysis. Alpha-diversity metrics (Chao 1) for bacterial and fungal communities were estimated using the diversity plugin, whereas Nonmetric Multidimensional Scaling (NMDS) was employed to visualize beta diversity dissimilarities based on Jaccard distances across different treatments.

#### Soil metabolome analyses

A fresh soil sample, weighing 5 g, was placed in a centrifuge tube and mixed with 50 mL of a methanol-hexane-water (3:1:1) solution at 30℃ for 1 h. The mixture was then centrifuged at 12,000 rpm for 10 min, and the resulting supernatant was filtered through a 0.22 µm membrane before being transferred to a detection bottle for LC-MS analysis.

All samples were analyzed using the LC-MS system according to the manufacturer’s protocols. Chromatographic separations were performed on a Vanquish ultra-performance liquid chromatography (UPLC) system (Thermo Fisher Scientific, USA). An ACQUITY UPLC HSS T3 column (2.1 mm × 150 mm, 1.8 µm) (Waters, Milford, MA, USA) was employed for the reversed-phase separation, with the column oven maintained at 40℃. The flow rate was set at 0.25 mL/min, and the injection volume at 2 µL. The mobile phase consisted of solvent A (water, 0.1% formic acid) and solvent B (acetonitrile, 0.1% formic acid) (43).

#### Co-occurrence network analyses

The underlying co-occurrences among bacterial and fungal taxa were depicted through network analysis using the CoNet plug-in in Cytoscape (44). Bacterial or fungal ASVs that occurred in fewer than 20% (45) of all 48 samples and had a sum relative abundance of less than 0.01% (46) in all samples were removed from the network analysis. Four networks were constructed based on all soil types, including black soil and meadow soil, for each treatment to ascertain the differences in microbiome composition of modules between control and fertilization treatments.

To investigate all the pairwise associations in co-occurrence network analyses, Spearman correlation and Kullback-Leibler dissimilarity (KLD) were computed. Subsequently, the dissimilarity threshold was set to the maximum value within the KLD matrix, whereas the Spearman’s correlation threshold was established at 0.7. To avoid potential false-positive correlations and compositionality biases, permutation and bootstrap distributions were generated with 1,000 iterations. The *P* values derived from these distributions were combined using Brown’s method (47). Following this integration, the Benjamini-Hochberg procedure was applied to control the false discovery rate (FDR), ensuring that the probability of falsely rejecting the null hypothesis did not exceed 0.05 (48). In the constructed networks, nodes represent amplicon sequence variants (ASVs), and edges signify strong and significant correlations between ASVs. Network topology parameters were calculated using the Network Analyzer plugin in Cytoscape. The resulting correlations were subsequently imported into the Gephi platform for visualization via the Fruchterman-Reingold algorithm.

#### Statistics analysis

A general linear model (GLM) was employed to analyze the effects of organic fertilizer, soil type, and their interaction on soil physicochemical properties, above- and below-ground biomass, phosphorus content, accumulations, and alpha diversity of the soil microbiome. Additionally, normality and variance heterogeneity/homogeneity were evaluated. One-way ANOVA with Duncan’s multiple range test was utilized to assess significant differences in each parameter among all treatments. The significance of the effects of organic fertilizer, soil type, and their interaction on differentiating soil bacterial and fungal communities was assessed through permutation multivariate analysis of variance (PERMANOVA). According to Spearman’s correlations and Mantel tests, pairwise comparisons of soil physicochemical properties for differential microorganisms based on all fertilization treatments within a soil type were performed. Random forest analysis was utilized to assess the contributions of differential microorganisms to soil physicochemical properties, based on all fertilization treatments within a soil type. The statistical difference threshold was set at *P* ≤ 0.05, and the variable importance for the projection (VIP) threshold was set at ≥1.0 for identifying the differential soil metabolites for pairwise or multiple groups. Agglomerative hierarchical clustering analysis was applied to soil differential metabolites among fertilization treatments within a soil type and visualized in a heatmap. Moreover, pairwise comparisons of the relative abundance of differential metabolites between fertilization treatments and the control within a soil type were analyzed using ANOVA. Spearman’s correlation was used to assess the correlation of differential microorganisms with soil differential metabolites based on all fertilization treatments within a soil type. Finally, a structural equation model (SEM) was used to explore the importance of organic manures on the changes in bacterial and fungal communities, phosphatase activity, differential metabolites, available phosphorus, and microbial biomass phosphorus in soils using SPSS-Amos (v26.0, IBM). The above analyses were performed and visualized using R 3.3.1, SPSS 16.0, and the Origin2024b platform, with statistical significance determined based on a *P*-value threshold of ≤0.05.

## RESULTS

### Organic manure physicochemical properties, phosphatase activity, and microbial community composition

The manure treated via traditional composting remained incompletely decomposed, characterized by high moisture content, uneven texture distribution, a dense structure, and persistent foul odor (Fig. S1). In contrast, the manure subjected to hyper-thermophilic fermentation with *Bacillus* strains inoculation achieved complete maturity (100%), exhibiting uniform texture, a porous structure, and the absence of detectable odor. The concentration of AP, MBP, and TP in the fermented manure was significantly higher than that in the composted manure (Table S1). The concentrations of AP, MBP, and TP in the fermented manure were significantly higher than those in the composted manure (*P* < 0.001; Table S1). Moreover, hyper-thermophilic fermentation markedly reduced (*P* < 0.001) the AP concentration while substantially increasing (*P* < 0.001) the MBP concentration compared with raw manure without treatment. The alpha and beta diversity of the microbial communities varied across different organic manure (Fig. S2). By comparing the bacterial and fungal community compositions across the different organic manures, we found the manure treated with hyper-thermophilic fermentation combined with *Bacillus* strains inoculation not only enriched the relative abundance of the bacterial genus *Bacillus* but also promoted another keystone bacterial genus, *Thermobifida* (Fig. S3).

### Oat growth, soil physicochemical, and enzymatic properties

**Fig 1:**
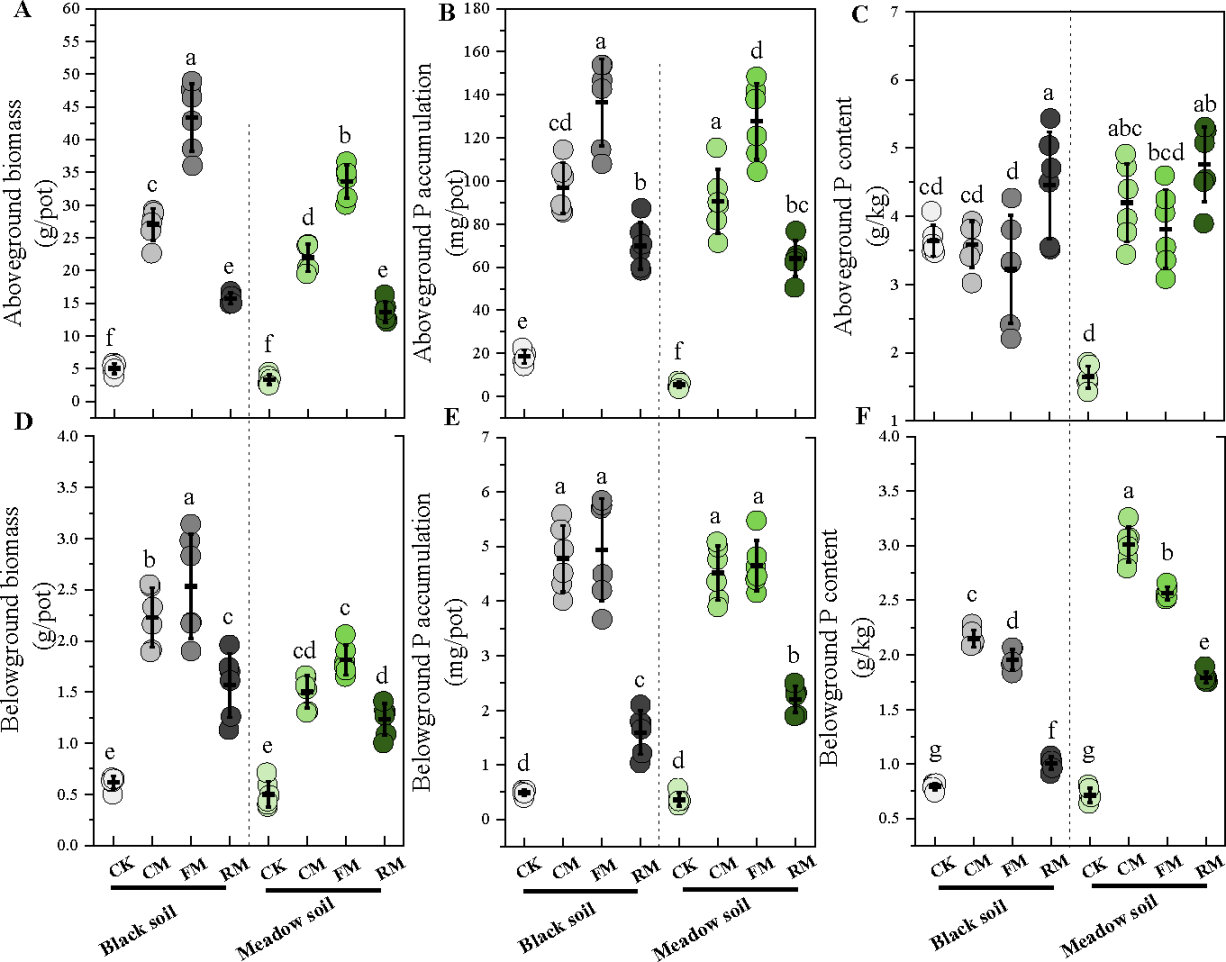
Effects of fertilization on oat above- and below-ground biomass (**A, D**), phosphorus accumulation (**B, E**), and phosphorus content (**C, F**). The differences in oat growth parameters among fertilization treatments within two soil types were tested by a one-way ANOVA (*P* < 0.05), and only significant differences observed in a comparison group were labeled with letters. Data represent mean ± SD (*n* = 6). CK: No manure addition; CM: Composted manure addition; FM: Fermented manure addition; and RM: Raw manure addition. Four fertilization treatments were applied to black soil and meadow soil, respectively, in a pot experiment.

The application of organic manure—composted manure, fermented manure, and raw manure—significantly influenced oat growth parameters. These parameters included both above-ground and below-ground biomass, as well as phosphorus content and accumulation (*P* < 0.001; Table S6). Compared with the control with no fertilization, the addition of the three types of organic manure significantly enhanced oat biomass and phosphorus accumulation in both soil types (black soil and meadow soil). The most effective was composted manure, followed by raw manure and fermented manure (Fig. 1).

**Fig 2:**
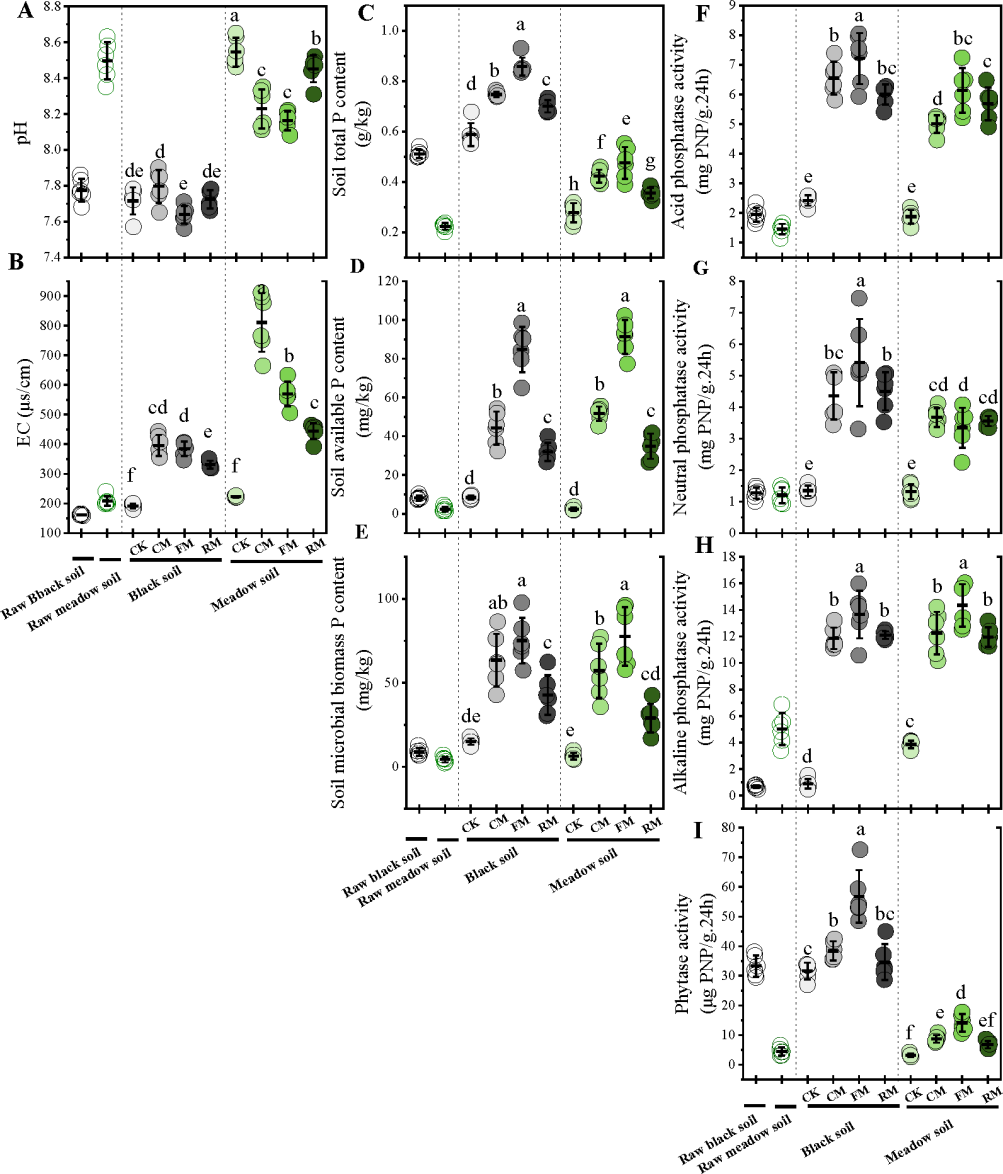
Effects of fertilization on soil physicochemical properties (**A-E**) and enzymatic activities (**F-I**). Differences in soil physicochemical properties among fertilization treatments within two soil types were tested by a one-way ANOVA (*P* < 0.05), and only significant differences observed in a comparison group were labeled with letters. Data represent mean ± SD (*n* = 6). CK: no manure addition; CM: Composted manure addition; FM: fermented manure addition; and RM: raw manure addition. Four fertilization treatments were applied to black soil and meadow soil, respectively, in a pot experiment.

Significant differences were observed in most soil physicochemical and enzymatic properties between the two types of soil (*P* < 0.01; Table S6). Black soil exhibited a more neutral pH, averaging 7.7 across all fertilization treatments, whereas meadow soil was weakly alkaline with an average pH of 8.5. TP in black soil was significantly higher (*P* < 0.05), although the differences in AP and MBP between the soils were not statistically significant. Enzymatic properties, particularly phytase activity, were significantly higher in black soil compared to meadow soil (*P* < 0.05), whereas alkaline phosphatase activity showed the opposite trend. There were also significant differences in soil physicochemical and enzymatic properties among the different fertilization treatments—including no fertilization, composted manure, fermented manure, and raw manure fertilization (*P* < 0.001; Table S6). The use of organic manures tended to lower soil pH in both soil types, indicating a trend toward acidification (Fig. 2). Relative to no fertilization, all organic manure applications significantly increased soil TP, AP, MBP, and enzymatic activities in both soil types, with the most pronounced effects observed in plots treated with fermented manure.

### Soil rhizosphere microbial community

#### Bacterial community

**Fig 3:**
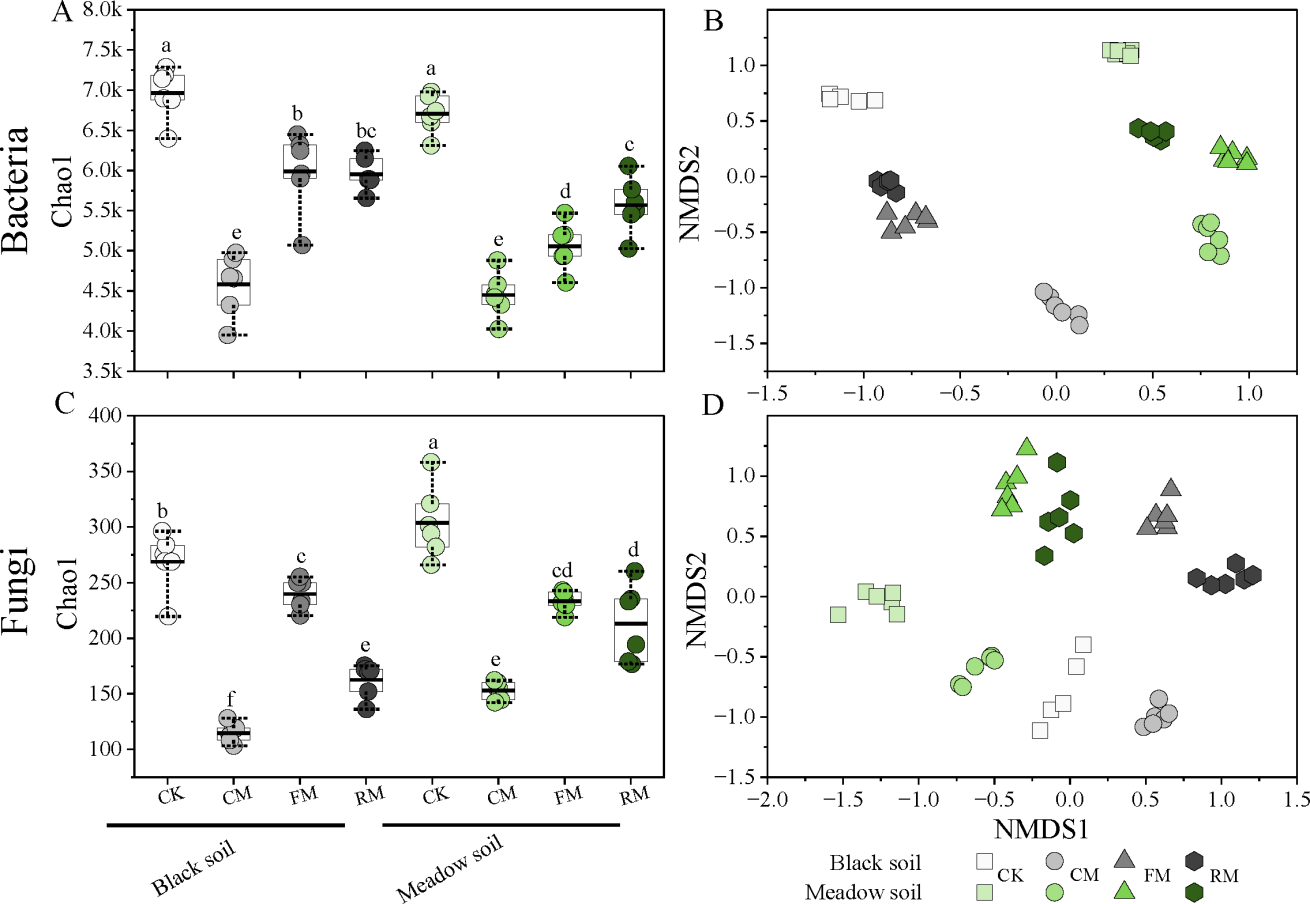
Alpha and beta diversity of the bacterial (**A, C**) and fungal (**B, D**) community in rhizosphere soil. The alpha diversity metric was estimated by Chao 1 at the ASV level. The difference in alpha diversity among fertilization treatments within two soil types was tested by a one-way ANOVA (*P* < 0.05), and only significant differences observed in a comparison group were labeled with letters. Data represent mean ± SD (*n* = 6). Beta diversity was analyzed by nonmetric multidimensional scaling (NMDS) based on Jaccard distance metrics at the ASV level and visualized in a scatter diagram. CK: No manure addition; CM: Composted manure addition; FM: Fermented manure addition; and RM: Raw manure addition. Four fertilization treatments were applied to black soil and meadow soil, respectively, in a pot experiment.

Organic manure fertilization significantly reduced the alpha diversity of the bacterial community in both soil types (Fig. 3A), although there were no overall differences in alpha diversity between black soil and meadow soil, except when fermented manure was used. The beta diversity of bacterial communities exhibited significant variations among the different fertilization treatments within the two soils (Fig. 3B).

**Fig 4:**
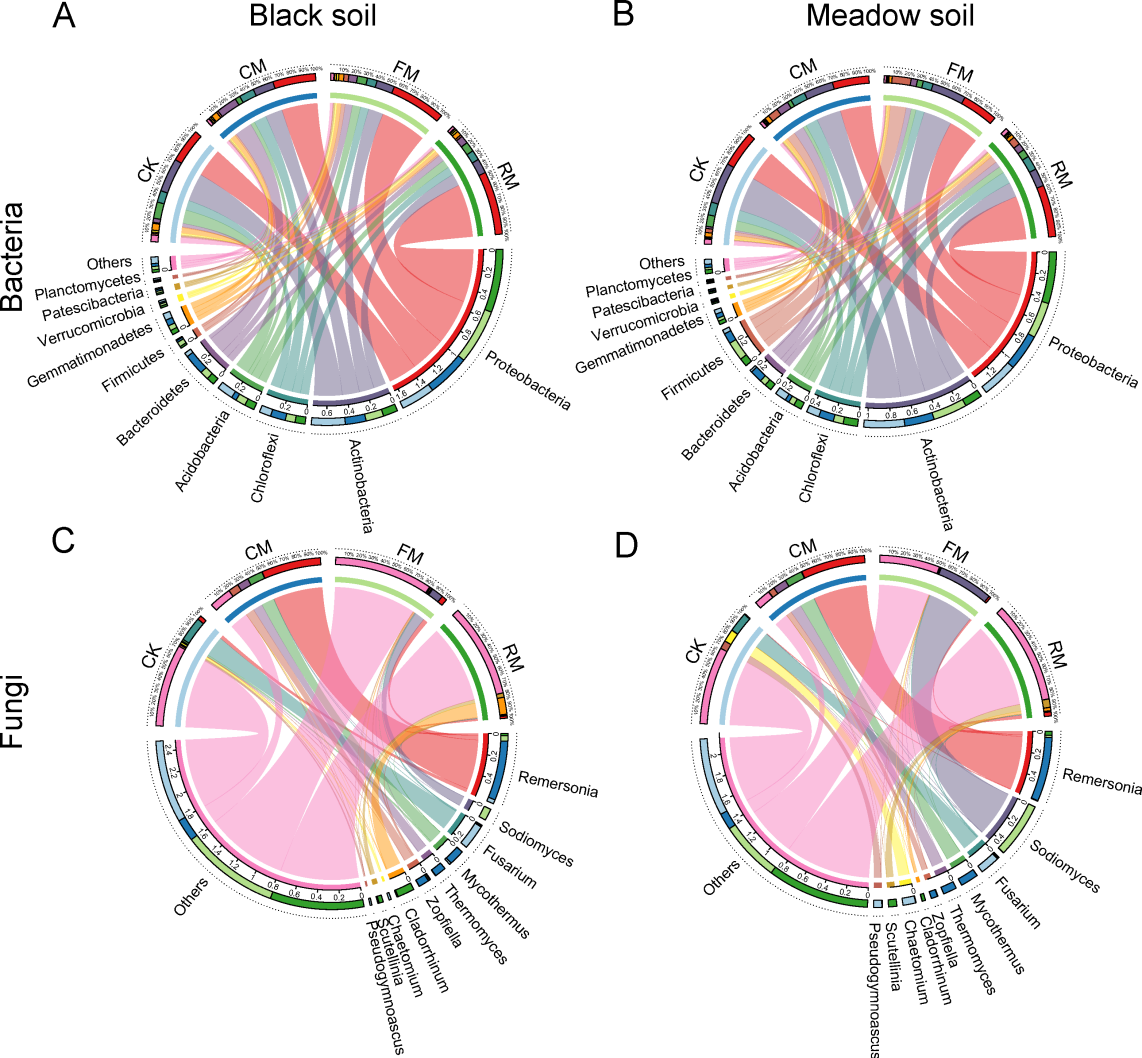
Relative abundance of taxonomic composition of soil bacterial (**A, B**) and fungal (**C, D**) communities at the phylum and genus levels, respectively, among four fertilization treatments within two soil types. CK: No manure addition; CM: Composted manure addition; FM: Fermented manure addition; and RM: Raw manure addition. Four fertilization treatments were applied to black soil and meadow soil, respectively, in a pot experiment.

The relative abundance of different bacterial phyla under various fertilization treatments in the two soil types is illustrated in Fig. 4A and B. In black soil, the relative abundance of Proteobacteria increased following organic manure fertilization and particularly with raw organic manure in meadow soil. Conversely, Chloroflexi increased only with composted manure fertilization in both soil types (*P* < 0.05; Fig. S7A and C). Actinobacteria and Acidobacteria significantly decreased in relative abundance across both soil types in response to organic manure fertilization (Fig. S7B and D ). Firmicutes were found in greater abundance in meadow soil than in black soil across all fertilization treatments (*P* < 0.05). However, Bacteroidetes and Gemmatimonadetes displayed opposite trends (*P* < 0.05), except with raw manure fertilization (Fig. S7E through G). Additionally, the application of fermented manure significantly increased the relative abundance of Firmicutes compared with other fertilizations, whereas the composted manure significantly boosted the relative abundance of other groups in both soils (*P* < 0.05).

At the genus level, the relative abundance of the bacterial community under all fertilization treatments in both soil types is depicted in Fig. S6A. Thermobifida, BIrii41, and Bacillus were most responsive to fermented manure fertilization, followed by composted and raw manure fertilizations in both soil types (Fig. S8C through F). The effects of fertilization on these bacterial genera varied between the soil types: *Thermobifida* and *Bacillus* were more abundant in meadow soil than in black soil, particularly under fermented manure conditions. In contrast, BIrii41 was found in lower abundance in meadow soil compared with black soil across all organic fertilizations. Additionally, SBR 1031 and JG30-KF-CM45 increased only with composted manure fertilization in both soil types, whereas *Devosia* increased with all types of organic manure fertilization only in black soil (*P* < 0.05; Fig. S8G through J).

#### Fungal community

Similar to the bacterial community, the alpha diversity of fungal communities was significantly affected by the application of organic manures (*P* < 0.05) (Fig. 3C). The beta diversity of these communities also exhibited dramatic variations among the different fertilization treatments within two soil types (Fig. 3D).

Among the top 10 fungal phyla, Ascomycota was predominantly found in all fertilization treatments across both soil types (Fig. S9). Specifically, the abundance of Ascomycota significantly decreased in treatments using organic manures, except for those involving composted manure, in both soil types (Fig. S9A). The phyla Basidiomycota and Mortierellomycota also significantly decreased across all organic manure treatments in both soil types. Conversely, the phyla Rozellomycota and Chytridiomycota significantly increased in raw manure treatments in black soil (Fig. S9B through E).

The relative abundance and taxonomic composition of the fungal community at the genus level across all fertilization treatments within the two soil types are depicted in Fig. 4C and D. Among the top 10 fungal genera, specific genera were enriched in distinct fertilization treatments (no fertilization, composted manure, fermented manure, and raw manure), and this pattern was consistent across both soil types (Fig. S10). More specifically, compared with plots without fertilization, those with composted manure saw significant increases in the relative abundance of Remersonia, Thermomyces, Mycothermus, and Zopfiella, whereas fermented manure treatments significantly increased the abundance of Sodiomyces in both soil types (Fig. S10A through F). Additionally, raw manure treatments significantly enhanced the abundance of Cladorrhinum and Scutellinia in both soil types (Fig. S10G through I). However, the relative abundance of Fusarium, Chaetomium, and Pseudogymnoascus was significantly lower in all organic manure treatments compared to no fertilization (Fig. S10C, H through J).

### Co-occurrence network between soil microorganisms

**Fig 5:**
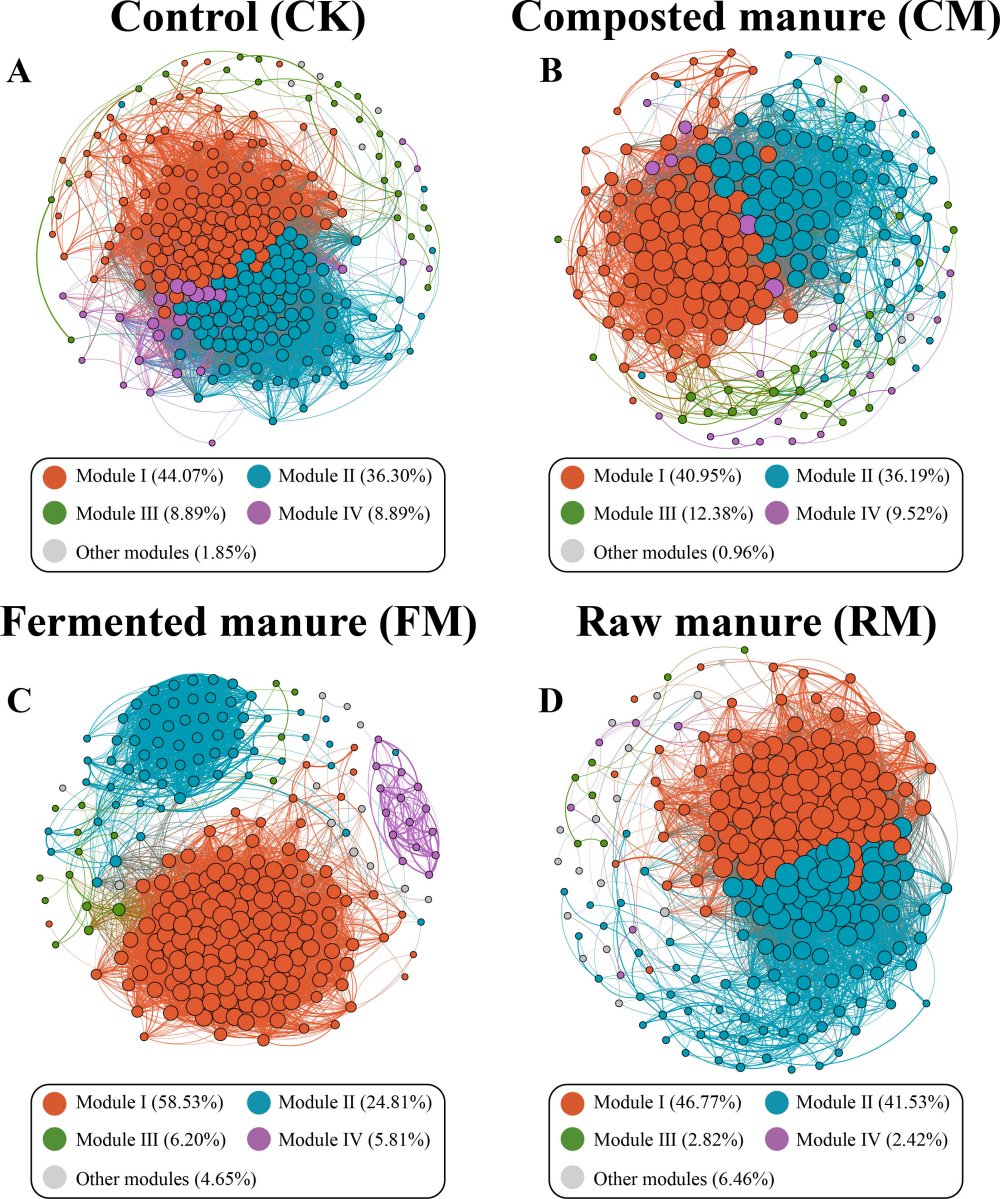
The networks visualize the effects of fertilization treatment (including no manure addition [CK], composted manure addition [CM], fermented manure addition [FM], and raw manure addition [RM]) on the co-occurrence pattern between bacterial and fungal taxa at the ASV level in soils. The networks in (**A–D**) were constructed based on fertilization treatment for all soil types together. The size of each node is proportional to the number of ASV links, and the nodes filled in blue are bacterial ASV and in yellow are fungal ASV. Networks are randomly colored by module.

To assess the general effects of organic manure fertilizations on soil microbiome associations, four networks were constructed for the different fertilization practices (composted manure, fermented manure, and raw manure) by combining all microbiomes from the two soil types (Fig. 5). The complexity of these networks, as indicated by the number of edges, nodes, and density, varied between the organic manure fertilizations and no manure fertilization. Networks resulting from organic manure fertilizations were less complex than those from no manure fertilization, with the network from composted manure fertilization showing the most significant differences, followed by fermented manure fertilization (Table S7). These networks displayed all modules with a node count exceeding 2% of the total nodes in each network. In these networks, Modules I and II constituted 80.37%, 77.14%, 83.34%, and 88.30% of the total nodes for no manure, composted manure, fermented manure, and raw manure fertilizations, respectively (Fig. 5A through D). We analyzed the phylogenetic composition of Modules I and II in each network, finding that their microbial phylum-level composition generally varied between networks from no manure fertilization and those from organic manure fertilizations (Fig. S11). The application of organic manure fertilization reduced the dominance of the fungal phyla Ascomycota and Basidiomycota and increased the dominance of the bacterial phyla Proteobacteria, Firmicutes, and Bacteroidetes in Modules I and II compared with no manure fertilization.

### Soil rhizosphere metabolites

In black soil, researchers identified a total of 78 differential metabolites, with 57 being upregulated, between composted manure and no manure fertilization. Similarly, 56 differential metabolites (44 upregulated) were identified between fermented manure and no manure fertilizations, and 54 differential metabolites (46 upregulated) were observed between raw fermented manure and no manure fertilizations (refer to Fig. S12A through C). In meadow soil, the numbers were 72 differential metabolites (59 upregulated) between composted manure and no manure fertilizations, 54 (45 upregulated) between fermented manure and no manure fertilizations, and 61 (46 upregulated) between raw fermented manure and no manure fertilizations (refer to Fig. S13A through C). Additionally, hierarchical clustering analysis utilizing Pearson correlation and Ward’s linkage effectively distinguished each fertilization treatment across both soil types. Heatmaps illustrate the expression profiles, showing the mean concentrations of all differential metabolites: 78 in total for black soil (Fig. S12D) and 81 in total for meadow soil (Fig. S13D). The analysis of differential metabolites revealed that the majority were upregulated in each organic manure fertilization compared with the control (no manure) in both soil types.

**Fig 6:**
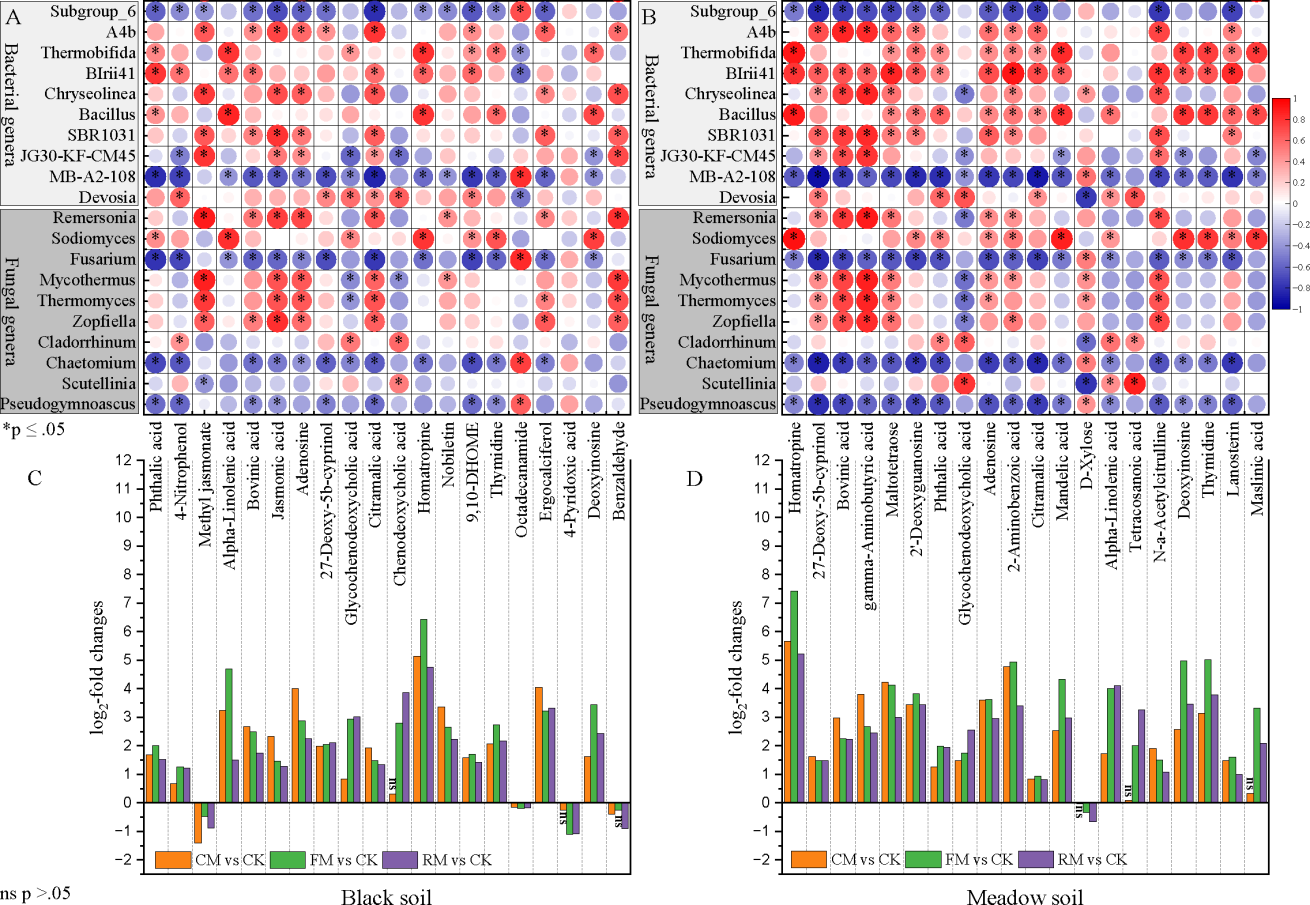
Correlation between differential microorganisms and differential metabolites. Pairwise comparisons of differential microorganisms (bacterial genera and fungal genera) with differential metabolites based on all fertilization treatments in black soil (**A**) and meadow soil (**B**), respectively. The colors of circles linking microorganisms to metabolites, indicating Spearman’s correlation coefficient; positive correlations are labeled with red and negative correlations are colored in blue. The significant correlations (*P* < 0.05) between differential microorganisms and differential metabolites were marked with asterisks. The differences in log_2_-fold changes (LFCs) in differential metabolites in organic manure fertilization treatments relative to CK treatment in black soil (**C**) and meadow soil (**D**), respectively. “ns” on the histogram indicates the insignificant difference between organic manure fertilization treatments and control treatment. CK: No manure addition; CM: Composted manure addition; FM: Fermented manure addition; and RM: Raw manure addition. Four fertilization treatments were applied to black soil and meadow soil, respectively, in a pot experiment.

Among all the differential metabolites identified across the four fertilizations within each soil type, the 20 most significantly differential metabolites are displayed in Fig. 6C and D. The majority of these metabolites were elevated in levels between organic manure and no manure fertilizations in both soil types. Notably, important organic acids with P-solubilizing functions, such as jasmonic acid and citramalic acid, were detected in black soil, and citramalic acid and mandelic acid were identified in meadow soil. Metabolic pathway enrichment analysis was conducted to evaluate differences in pathways between each organic manure fertilization and no manure fertilization. The results revealed the 20 most significantly enriched pathways influenced by differential metabolites between each organic manure fertilization (composted, fermented, or raw manure) and no manure fertilization in both black soil (Fig. S14 A, C, and E) and meadow soil (Fig. S15 A, C, and E). The majority of these pathways were upregulated, particularly between fermented manure fertilizations and no manure fertilization in both black soil (Fig. S14 B, D, and F) and meadow soil (Fig. S14 B, D, and F).

### Correlation among differential microorganisms, metabolites, and environmental factors

**Fig 7:**
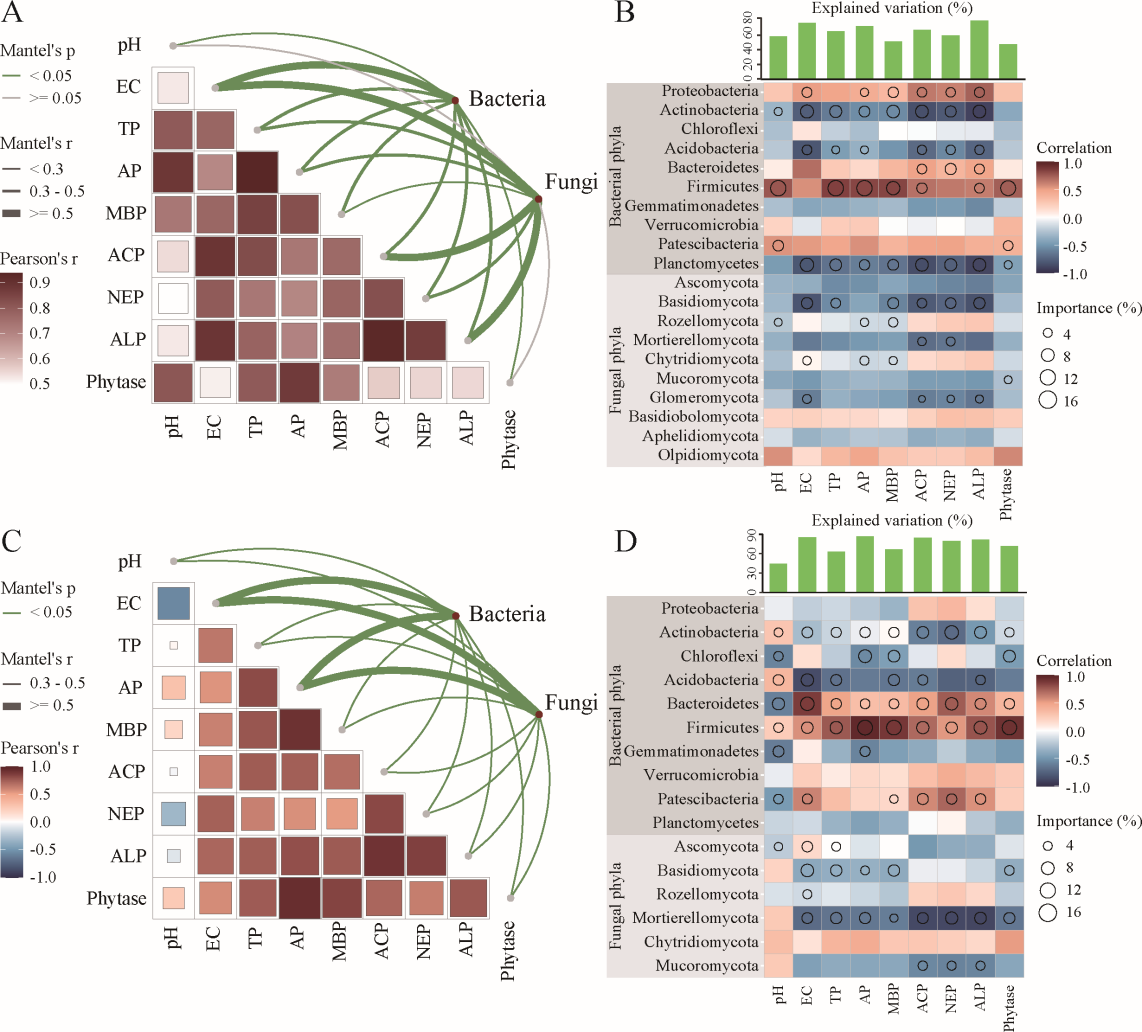
Correlation between differential microorganisms and environmental factors. Pairwise comparisons of environmental factors with bacterial and fungal communities in black soil (**A**) and meadow soil (**C**), respectively, with a color gradient denoting Spearman’s correlation coefficient. Edge width corresponds to Mantel’s r statistic for the corresponding distance correlations, and edge color denotes the statistical significance based on 9,999 permutations. Random forest analysis of environmental factors and differential microorganisms (bacterial phyla and fungal phyla) in a black soil (**B**) and meadow soil (**D**), respectively. Circle size represents the variable’s importance. Colors represent Spearman’s correlations. The total explanatory power of the differential microorganisms on environmental factors is displayed as a histogram above the heatmap. TP: total phosphorus; AP: available phosphorus; MBP: microbial biomass phosphorus; ACP: acid phosphatase; NEP: neutral phosphatase; and ALP: alkaline phosphatase.

Soil physico-chemical properties (TP, AP, MBP, ACP, NEP, and ALP) strongly predicted the dissimilarities in bacterial and fungal community compositions in both black and meadow soils (Fig. 7A and C; Tables S8 and S10). Notably, the bacterial phyla Firmicutes and Bacteroidetes were identified as the most significant universal drivers influencing all soil physico-chemical properties, particularly TP, AP, MBP, and phytase in both soil types (Fig. 7B and D; Tables S9 and S11). Additionally, the bacterial genera *Thermobifida*, BIrii41, and *Bacillus*, along with the fungal genus *Sodiomyces*, were key taxa responsible for changes in soil physico-chemical properties in both types of soil (Fig. S16; Tables S12 and S13). Furthermore, these microorganisms showed a positive correlation with most rhizosphere metabolites in both soil types (Fig. 6A and B).

### Influential factors on soil available phosphorus and microbial biomass phosphorus

**Fig 8:**
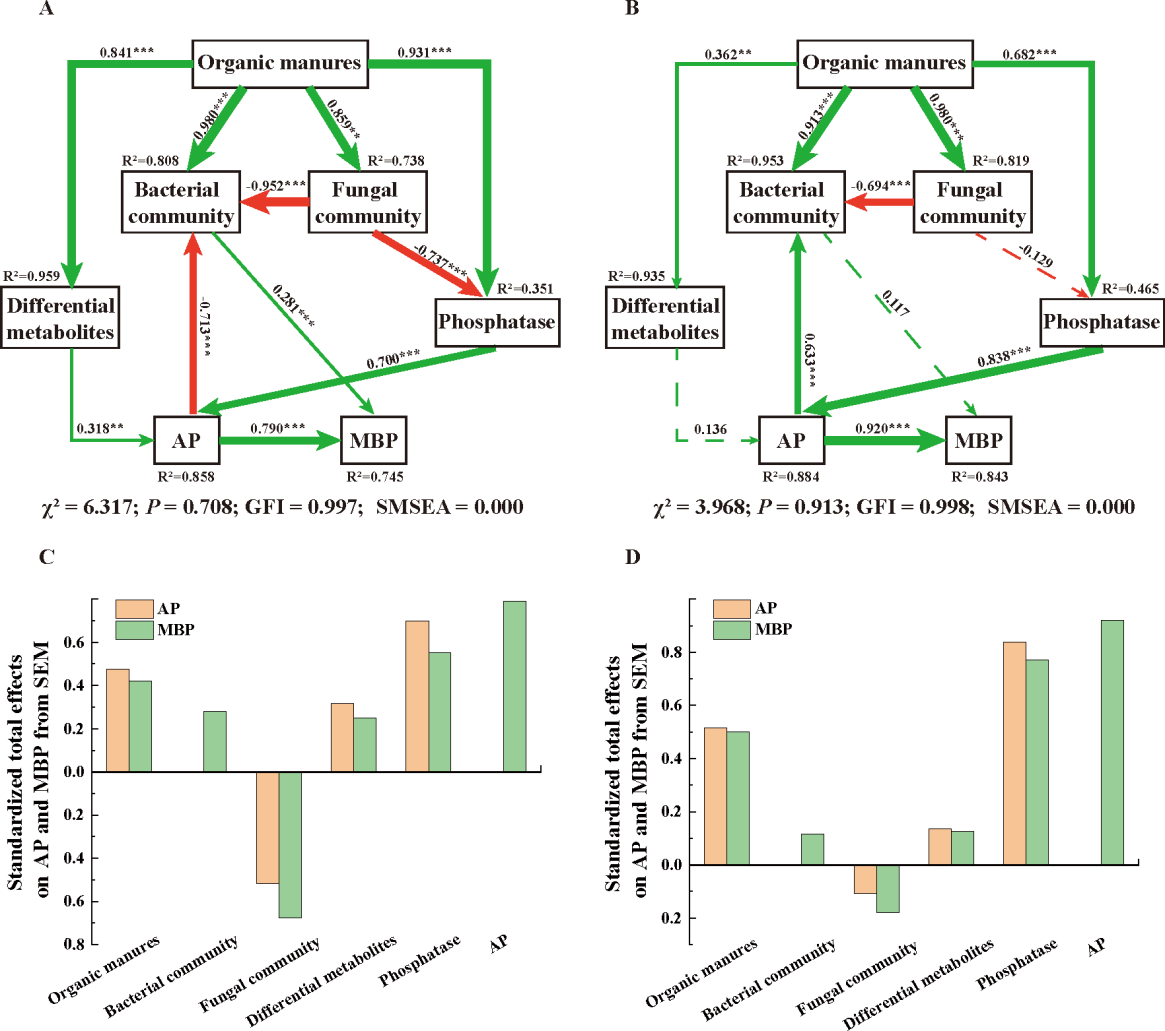
Structural equation model (SEM) showing the direct and indirect effects of organic manure fertilization treatments on soil available phosphorus (AP) and microbial biomass phosphorus (MBP) based on the results of GLM analysis in black soil (**A**) and meadow soil (**B**), respectively. Continuous and dashed arrows represent significant and nonsignificant relationships between variables, respectively, and green and red arrows indicate positive and negative relationships, respectively. Adjacent numbers that are labeled in the same direction as the arrow are path coefficients, and the width of the arrow is proportional to the degree of path coefficients. R^2^ values indicate the proportion of variance explained for each variable. Significance levels are denoted with *P < 0.05, **P < 0.01, ***P < 0.001. Standardized total effects (direct plus indirect effects) calculated by the SEMs (**A, B**) are displayed in **C, D**, respectively, below the SEMs. The low (χ2), nonsignificant probability level (*P* > 0.05), high goodness-of-fit index (GFI > 0.90), low Akaike information criteria (AIC), and low root-mean-square errors of approximation (SMSEA < 0.05) listed below the SEMs indicate that our data matches the hypothetical models

SEMs (Fig. 8) quantified the contributions of various potential influential factors (including organic manures, bacterial and fungal communities, differential metabolites, and phosphatases) to the significant increase (*P* < 0.05) in soil AP and MBP following the application of organic manures in different soil types. Differential metabolites and phosphatases directly affected soil AP, with phosphatases contributing more to the increases in soil AP in black soil (0.700) and in meadow soil (0.838). Additionally, among all the direct influential factors on soil MBP, soil AP had a more direct impact on soil MBP in black soil (0.790) and meadow soil (0.920) than the bacterial communities in these two soil types.

### Quantification of oat phosphorus uptake rate, residual rate, and loss rate from organic manures

**Fig 9:**
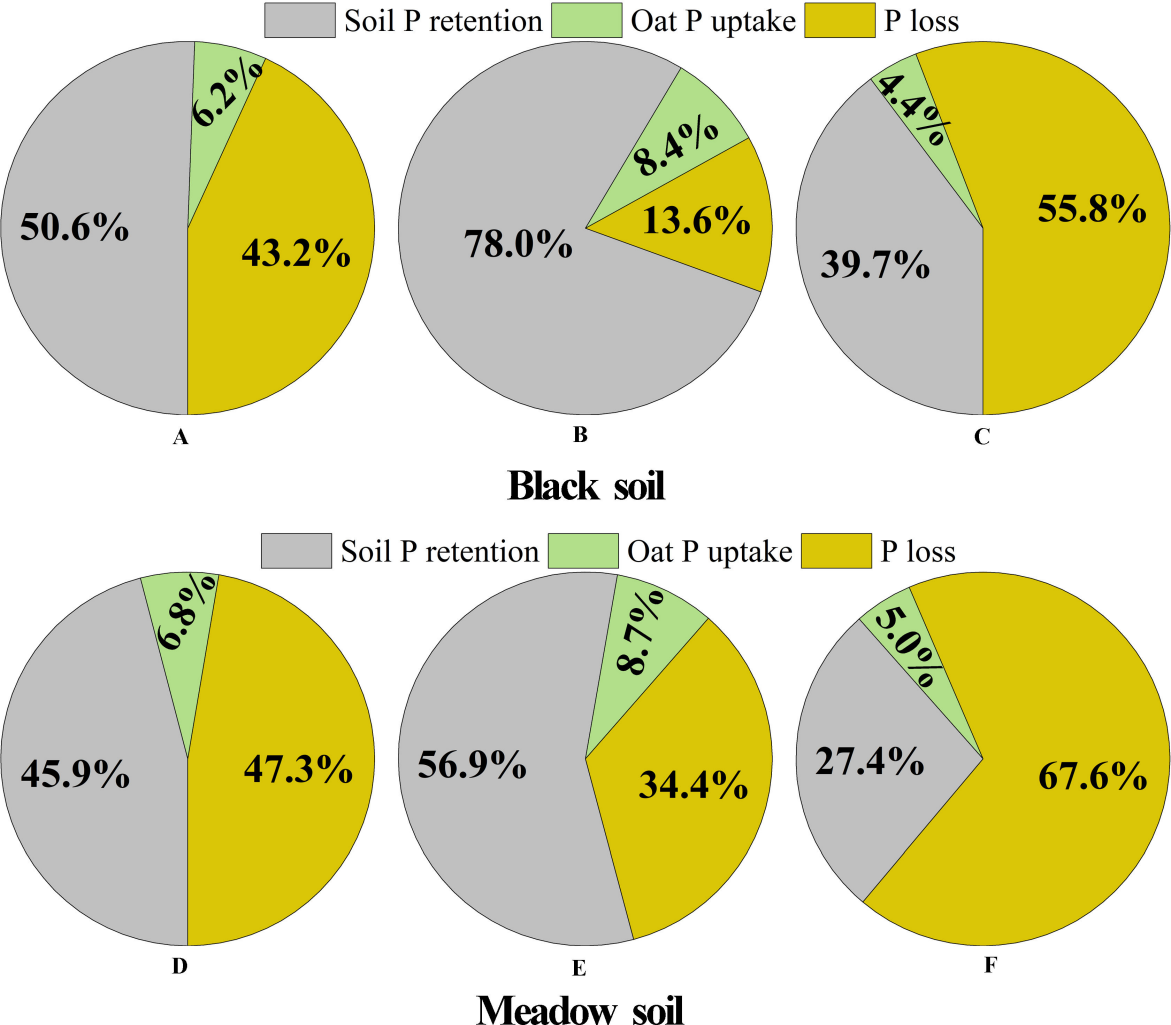
Phosphorus transfer from organic manure to oats and soils under different fertilization treatments. Quantification of phosphorus transfer in organic manure, including the oat phosphorus uptake, soil phosphorus retention, and phosphorus loss among different fertilization treatments in black soil (**A–C**) and meadow soil (**D–F**), respectively. A-C represent the composted manure addition, the fermented manure addition, the raw manure addition, respectively, in black soil. B-F represent the composted manure addition, the fermented manure addition, the raw manure addition, respectively, in meadow soil. Plant and soil samplings were conducted during the 12th week (the end of the oat growth period) after the application of organic manure in a pot experiment.

The transfer of phosphorus from organic manure to oats and soils varied significantly depending on the type of organic manure used. Fermented manure exhibited superior performance relative to conventional composted manure, as it increased phosphorus uptake rate of oats by 35.5% in black soil and 27.9% meadow soil, respectively, over a single growing season (Fig. 9). Additionally, after the application of fermented manure, its own majority of phosphorus was retained in soils, thereby mitigating the risk of phosphorus loss, particularly when applied to black soil. In the case of fermented manure, the soil phosphorus retention rates were highest at 78.0% and 56.9%, and the phosphorus loss rates were lowest at 13.6% and 34.4% of total phosphorus when applied to black soil and meadow soil, respectively (Fig. 9B and E). In contrast, raw manure was the least effective, retaining only a small portion of its phosphorus in the soil, with more than half of its phosphorus lost. The soil phosphorus retention rates were lowest at 39.7% and 27.4%, and the phosphorus loss rates were highest at 55.8% and 67.6% of phosphorus in raw manure when applied to black soil and meadow soil, respectively (Fig. 9C and F). Composted manure showed relatively better performance, with soil phosphorus retention and loss rates falling between those of fermented and raw manure (Fig. 9A and D).

## DISCUSSION

We found that the application of organic manures altered the soil’s physico-chemical properties, particularly increasing soil phosphorus availability, with differences depending on the soil type. We revealed that organic manure fertilization-induced changes, such as rhizosphere acidification and increased phosphatase activities, directly enhanced the availability of soil phosphorus, which varied slightly by soil type. This aligns with a previous study that showed organic acids and phosphatases correlated with soil labile and moderately labile phosphorus pools (49). In this study, the application of organic manure significantly upregulated the majority of differential metabolites, most of which belong to organic acids and derivatives, particularly including representative organic acids such as jasmonic acid and citramalic acid detected in black soil, and citramalic acid and mandelic acid detected in meadow soil. These organic acids have a high capacity to solubilize insoluble P and thus have received widespread attention (50, 51). Additionally, organic manure fertilization also significantly increased the activities of ACP, NEP, ALP, and phytase, with the effect being much stronger in black soil compared to that in meadow soil (except for ALP). This is consistent with the typical theory that ACP and ALP activities are sensitive to pH changes, being negatively affected for ACP activity and positively for ALP activity (52). Other studies have also revealed that organic manure fertilizations significantly promoted the average concentrations of ACP and ALP, activating organic P (53, 54). We further reveal that fermented manure greatly increases soil phosphorus availability compared with the application of raw or composted manure, which could be attributed to greater rhizosphere acidification and phosphatase activities.

Fertilization management regimes can affect soil properties and soil microbial diversity (55). In this study, we also found that the oat rhizosphere microbiome was sensitive to the application of organic manures, as bacterial and fungal communities underwent significant changes and their diversities were notably reduced, with consistencies observed in two soil types. Previous studies have shown that the application of organic manures either increased microbial diversity (56), decreased it (57), or decreased only the bacterial alpha diversity without affecting the fungal alpha diversity (58). These findings indicate an inconsistent response of the soil microbial community, which may be determined by organic manure types, fertilization practices, soil types, and crops, among other factors. The constructed network graphs visualized different structures for microbial communities in the oat rhizosphere with organic manures compared with control treatments. The decreases in the node degrees and edges of the microbial co-occurrence network showed that the application of organic manures weakened the complexity of networks, consistent with previous studies that demonstrated a 7-year organic manure application reduced competition and network complexity among rhizosphere microbial communities (59). Additionally, the microbial community in the oat rhizosphere treated with organic manure appeared more organized and simplified, particularly in modules I and II, compared to those under no organic manure treatment. Given that highly connected microorganisms within a module often co-occur and may share similar interrelated functions within communities (60), our results suggest that soils treated with organic manure could harbor specialized ecologically functional groups. Previous research by (61) showed that high nutrient additions could promote some competitively strong taxa that outcompete others, thus simplifying microbial communities and causing irreversible changes. We hypothesize that the addition of exogenous nutrients increases resource and food availability, which subsequently alters the microbial community to enhance the efficiency of resource turnover, ultimately benefiting plant growth.

Inorganic fertilizer input is a key driver for the distribution of active PSMs (62). Long-term use of high inorganic phosphorus fertilizer has increased the relative abundance of soil phosphate-solubilizing genera such as *Arthrobacter*, *Bacillus*, and *Flavobacterium*, which are important predictors of soil AP. This has consequently reshaped the microbial community structure in agricultural soils (63, 64). Similarly, long-term application of organic manure has also increased PSM populations in the microbial communities of various agricultural soils (65, 66). Organic manures, rich in both organic and inorganic phosphorus, provide significant amounts of exogenous phosphorus to the soil, which can be further concentrated through composting or fermentation (67, 68). In this study, we identified the typical phosphate-solubilizing genus Bacillus; however, it had an extremely low relative abundance in control soils. Moreover, the application of composted organic manure only weakly increased the relative abundance of *Bacillus*. The minor or non-significant effect of composted manure application on these PSMs may be due to the relatively short term of fertilizer application, as fertilization-induced biotic changes often only become apparent after several years (69). Additionally, traditional composted organic manure typically has lower phosphorus availability compared with chemical phosphorus fertilizers, resulting in P-poor habitats in the short term, which are unfavorable for copiotrophic PSMs (66).

Making full and effective use of PSMs to mobilize previously unavailable forms of soil P for plants has become a prominent research topic in the fields of plant nutrition and ecology (70). Introducing microbial inoculants is a simple yet effective approach for functionally targeted modulation of the rhizosphere microbiome to enhance plant fitness. Previous studies have reported that PSM inoculation increased crop dry weight, phosphorus uptake, and soil available phosphorus and decreased the content of moderately labile phosphorus and stable phosphorus, compared with controls (50, 71). However, inoculation of the rhizosphere microbiome often results in suboptimal or transient colonization, due to a variety of factors that influence the fate of the inoculant (61). In the present study, we innovatively inoculated several heat-resistant phosphate-solubilizing *Bacillus* strains into raw manure and fermented it in a hyper-thermophilic reactor to promote the enrichment of the *Bacillus* strains. Consequently, we utilized heat-resistant phosphate-solubilizing Bacillus during hyper-thermophilic fermentation to specialize in promoting phosphorus transformation and producing phosphorus-enriched organic manure. Subsequently, this fermented manure, in contrast to raw manure and traditional composted manure, was then applied to black soil and meadow soil to evaluate the oat rhizosphere microbial community composition and phosphorus transformation.

We found that this technique not only significantly increased the relative abundance of the bacterial genus *Bacillus* but also bacterial genus *Thermobifida* in fermented manure, which was scarcely detectable in composted and raw manure. This result indicates that the introduced heat-resistant PSMs exhibited great competitiveness and successfully established dominance in fermented manure under an ultra-high temperature environment. Interestingly, the extremely low abundance of the hyper-thermophilic bacterial genus *Thermobifida* in raw and composted manure also became dominant in fermented manure. Our results further indicate that thermophilic microorganisms can grow normally and eventually dominate under ultra-high temperature conditions. This is consistent with previous studies that have shown that with the increase in fermentation temperature, the microbial community experiences a shift from mesophilic to hyper-thermophilic bacteria (26, 72). Compared with raw and composted manure, the fermented manure exhibited a significantly higher total phosphorus concentration, indicating that hyper-thermophilic fermentation is more effective in enriching phosphorus in organic manure. More importantly, hyper-thermophilic fermented manure contained a higher concentration of microbial biomass phosphorus, which represents phosphorus with high biological activity and can be rapidly converted into plant-available forms when crops require. Furthermore, when hyper-thermophilic fermented manure was applied, the results showed that these microorganisms became abundant in the oat rhizosphere across both soil types, with their presence being significantly higher compared with other organic manure fertilizations. This is a valuable success, as previous studies have revealed that most exogenous microorganisms from organic manure do not survive in soil conditions for more than a few months after being introduced (56, 73). Random forest analysis indicated that *Bacillus* and *Thermobifida* were most positively related to soil physicochemical properties, particularly TP, AP, and MBP. Based on these findings, we assumed that the differential soil microorganisms *Bacillus* and *Thermobifida* appeared as universal key driving factors and also exhibited a synergistic effect in phosphorus transformation, given their recognized roles in phosphorus solubilization and cellulolytic degradation, respectively (74, 75). Additionally, other beneficial biomarkers such as the bacterial phylum Firmicutes, the bacterial genus BIrii41, and the fungal genus Sodiomyces were identified and greatly enriched in the oat rhizosphere under fermented manure application. It is generally believed that these microorganisms are regarded as plant growth-promoting microorganisms, and their presence was conducive to nutrient cycling (76–78). However, their specific underlying mechanisms for driving soil nutrient cycling could be further researched. Our results reinforce the potential of these microorganisms as promising indicators that should be prioritized in future efforts to be implemented in agricultural systems for phosphorus transformation.

Chemical phosphate fertilizers have traditionally been used to address phosphorus deficiency in agricultural production. However, their use has been constrained by the non-renewability of raw materials and negative impacts on ecological health (79, 80). Manure waste, as a renewable resource, provides an important source of phosphorus. PSMs play a crucial role in converting these abundant raw materials into valuable organic manure products (24, 81). Organic manure has become a mainstream fertilization method due to its benefits for soil health and stability. It enhances microbial phosphorus solubilization and mineralization, increasing phosphorus availability and promoting microbial phosphorus immobilization, thereby reducing environmental losses in agricultural soils (82, 83). In our study, we found that the fermented manure prepared by inoculating PSMs was more effective compared with other organic manures. It retained a substantial proportion of phosphorus in the soil—78.0% in black soil and 56.9% in meadow soil, respectively—resulting in minimal phosphorus loss of 13.6% and 34.4%, respectively.

Microorganisms can act as catalysts to drive a new circular bioeconomy in agricultural systems (84). This study introduces new concepts and techniques into both fundamental and applied research. Specifically, hyper-thermophilic fermentation with Bacillus strain inoculation not only increased the dominance of inoculated Bacillus and the rare Thermobifida in organic manure but also enhanced their survival and dominance in the oat rhizosphere when the fermented manure was applied to the soil. This maximizes the efficacy of PSMs as phosphorus activators. However, a deeper understanding of phosphorus mobilization by PSMs for plant phosphorus nutrition and the use of phosphorus-mobilizing inoculants as biofertilizers requires further research (70). Additionally, our results reveal that changes in soil physico-chemical properties and the oat rhizosphere microbiome are similar between soil types, despite significant differences in basic soil properties. This suggests that the effects of the organic manures used in this study should be tested in field trials across broader temporal and spatial scales, and also considering more crops and soil types to accurately assess the responses of the rhizosphere microbiome (85).

### Conclusions

We investigated the effects of four different fertilization treatments—no manure, composted manure, fermented manure, and raw manure—on the physico-chemical properties, microbiome, and metabolites of the rhizosphere. The use of organic manures increased soil TP, AP, and MBP concentrations and enhanced the activities of ACP, NEP, ALP, and phytase and also led to rhizosphere acidification, with fermented manure showing the greatest effects. SEMs revealed that organic manures indirectly influence soil phosphorus availability (TP, AP, and MBP) by inducing rhizosphere acidification and enhancing phosphatase activities in both investigated soil types. Random forest analysis indicated bacterial genera such as *Bacillus*, *Thermobifida*, and *BIrii41* were identified as universal key drivers that influenced all measured soil physico-chemical properties, especially soil AP and MBP. Moreover, *Bacillus* and *Thermobifida* may have a synergistic effect on phosphorus transformation, given their established roles in phosphorus solubilization and cellulolytic degradation, respectively. The relative abundance of these soil microorganisms was notably higher in the oat rhizosphere under fermented manure fertilization compared with other treatments, across both soil types. Therefore, the application of fermented manure proved to be superior, as it significantly enhanced the transfer of phosphorus from organic manure to oats and soils in both black and meadow soils.

## Data Availability

The datasets supporting the conclusions of this article are publicly available at https: https://doi.org/10.6084/m9.figshare.29665730. The 16S rRNA gene and ITS rRNA gene amplicon reads are deposited in NCBI under accession numbers PRJNA1225494 and PRJNA1225595, respectively.

## SUPPLEMENTAL MATERIAL

Supplemental material aem.01208-25-s0001.docx Tables S1 to S13; Fig. S1 to S16.

Graphical abstract aem.01208-25-s0002.tif Visual depiction of the study.

ASM does not own the copyrights to Supplemental Material that may be linked to, or accessed through, an article. The authors have granted ASM a non-exclusive, world-wide license to publish the Supplemental Material files. Please contact the corresponding author directly for reuse.

## References

[1] Nizami AS, Rehan M, Waqas M, Naqvi M, Ouda OKM, Shahzad K, Miandad R, Khan MZ, Syamsiro M, Ismail IMI, Pant D. 2017. Waste biorefineries: enabling circular economies in developing countries. Bioresour Technol 241:1101–1117. doi:10.1016/j.biortech.2017.05.097 28579178

[2] Foley JA, Defries R, Asner GP, Barford C, Bonan G, Carpenter SR, Chapin FS, Coe MT, Daily GC, Gibbs HK, Helkowski JH, Holloway T, Howard EA, Kucharik CJ, Monfreda C, Patz JA, Prentice IC, Ramankutty N, Snyder PK. 2005. Global consequences of land use. Science 309:570–574. doi:10.1126/science.1111772 16040698

[3] Zhang Z, Wei Z, Guo W, Wei Y, Luo J, Song C, Lu Q, Zhao Y. 2021. Two types nitrogen source supply adjusted interaction patterns of bacterial community to affect humifaction process of rice straw composting. Bioresour Technol 332:125129. doi:10.1016/j.biortech.2021.125129 33857866

[4] Luo G, Sun B, Li L, Li M, Liu M, Zhu Y, Guo S, Ling N, Shen Q. 2019. Understanding how long-term organic amendments increase soil phosphatase activities: Insight into phoD- and phoC-harboring functional microbial populations. Soil Biology and Biochemistry 139:107632. doi:10.1016/j.soilbio.2019.107632

[5] Köninger J, Lugato E, Panagos P, Kochupillai M, Orgiazzi A, Briones MJI. 2021. Manure management and soil biodiversity: towards more sustainable food systems in the EU. Agric Syst 194:103251. doi:10.1016/j.agsy.2021.103251

[6] Chojnacka K, Moustakas K, Witek-Krowiak A. 2020. Bio-based fertilizers: a practical approach towards circular economy. Bioresour Technol 295:122223. doi:10.1016/j.biortech.2019.122223 31623921

[7] Fu D, Wu X, Duan C, Zhao L, Li B. 2020. Different life-form plants exert different rhizosphere effects on phosphorus biogeochemistry in subtropical mountainous soils with low and high phosphorus content. Soil and Tillage Research 199:104516. doi:10.1016/j.still.2019.104516

[8] Yang X, Post WM. 2011. Phosphorus transformations as a function of pedogenesis: a synthesis of soil phosphorus data using Hedley fractionation method. Biogeosciences 8:2907–2916. doi:10.5194/bg-8-2907-2011

[9] Maltas A, Kebli H, Oberholzer HR, Weisskopf P, Sinaj S. 2018. The effects of organic and mineral fertilizers on carbon sequestration, soil properties, and crop yields from a long‐term field experiment under a Swiss conventional farming system. Land Degrad Dev 29:926–938. doi:10.1002/ldr.2913

[10] Tie J, Gao X, Liu Y, Chen W, Hu L, Yu J, Li T. 2024. Improving the value of planting and breeding waste compost in agricultural applications: a zucchini cultivation case and circular agricultural models analysis. Chem Eng J 496:153984. doi:10.1016/j.cej.2024.153984

[11] Nahidan S, Ghasemzadeh M. 2022. Biochemical phosphorus transformations in a calcareous soil as affected by earthworm, cow manure and its biochar additions. Agric, Ecosyst Environ, Appl Soil Ecol 170:104310. doi:10.1016/j.apsoil.2021.104310

[12] Yang X, Chen X, Yang X. 2019. Effect of organic matter on phosphorus adsorption and desorption in a black soil from Northeast China. Soil and Tillage Research 187:85–91. doi:10.1016/j.still.2018.11.016

[13] Angst TE, Sohi SP. 2013. Establishing release dynamics for plant nutrients from biochar. GCB Bioenergy 5:221–226. doi:10.1111/gcbb.12023

[14] Wu Q, Kwak J-H, Chang SX, Han G, Gong X. 2020. Cattle urine and dung additions differently affect nitrification pathways and greenhouse gas emission in a grassland soil. Biol Fertil Soils 56:235–247. doi:10.1007/s00374-019-01415-1

[15] Kong Y, Zhang J, Zhang X, Gao X, Yin J, Wang G, Li J, Li G, Cui Z, Yuan J. 2024. Applicability and limitation of compost maturity evaluation indicators: a review. Chem Eng J 489:151386. doi:10.1016/j.cej.2024.151386

[16] Meilander J, Caporaso JG. 2024. Microbiome science of human excrement composting. ISME J 18:228. doi:10.1093/ismejo/wrae228 PMC11631093 39520251

[17] Ajmal M, Shi A, Awais M, Mengqi Z, Zihao X, Shabbir A, Faheem M, Wei W, Ye L. 2021. Ultra-high temperature aerobic fermentation pretreatment composting: parameters optimization, mechanisms and compost quality assessment. J Environ Chem Eng 9:105453. doi:10.1016/j.jece.2021.105453

[18] Ge T, Wei X, Razavi BS, Zhu Z, Hu Y, Kuzyakov Y, Jones DL, Wu J. 2017. Stability and dynamics of enzyme activity patterns in the rice rhizosphere: effects of plant growth and temperature. Soil Biology and Biochemistry 113:108–115. doi:10.1016/j.soilbio.2017.06.005

[19] Sigurnjak I, Michels E, Crappé S, Buysens S, Tack FMG, Meers E. 2016. Utilization of derivatives from nutrient recovery processes as alternatives for fossil-based mineral fertilizers in commercial greenhouse production of Lactuca sativa L. Sci Hortic 198:267–276. doi:10.1016/j.scienta.2015.11.038

[20] Luo G, Ling N, Nannipieri P, Chen H, Raza W, Wang M, Guo S, Shen Q. 2017. Long-term fertilisation regimes affect the composition of the alkaline phosphomonoesterase encoding microbial community of a vertisol and its derivative soil fractions. Biol Fertil Soils 53:375–388. doi:10.1007/s00374-017-1183-3

[21] Liu X, Han R, Cao Y, Turner BL, Ma LQ. 2022. Enhancing phytate availability in soils and phytate-p acquisition by plants: a review. Environ Sci Technol 56:9196–9219. doi:10.1021/acs.est.2c00099 35675210 PMC9261192

[22] Wang Y, Luo D, Xiong Z, Wang Z, Gao M. 2023. Changes in rhizosphere phosphorus fractions and phosphate-mineralizing microbial populations in acid soil as influenced by organic acid exudation. Soil and Tillage Research 225:105543. doi:10.1016/j.still.2022.105543

[23] Efthymiou A, Grønlund M, Müller-Stöver DS, Jakobsen I. 2018. Augmentation of the phosphorus fertilizer value of biochar by inoculation of wheat with selected Penicillium strains. Soil Biol Biochem 116:139–147. doi:10.1016/j.soilbio.2017.10.006

[24] Raymond NS, Gómez-Muñoz B, van der Bom FJT, Nybroe O, Jensen LS, Müller-Stöver DS, Oberson A, Richardson AE. 2021. Phosphate-solubilising microorganisms for improved crop productivity: a critical assessment. New Phytol 229:1268–1277. doi:10.1111/nph.16924 32929739

[25] Jansson JK, McClure R, Egbert RG. 2023. Soil microbiome engineering for sustainability in a changing environment. Nat Biotechnol 41:1716–1728. doi:10.1038/s41587-023-01932-3 37903921

[26] Zhao Y, Liu Z, Zhang B, Cai J, Yao X, Zhang M, Deng Y, Hu B. 2023. Inter-bacterial mutualism promoted by public goods in a system characterized by deterministic temperature variation. Nat Commun 14:5394. doi:10.1038/s41467-023-41224-7 37669961 PMC10480208

[27] Chen W, Zhan Y, Zhang X, Shi X, Wang Z, Xu S, Chang Y, Ding G, Li J, Wei Y. 2022. Influence of carbon-to-phosphorus ratios on phosphorus fractions transformation and bacterial community succession in phosphorus-enriched composting. Bioresour Technol 362:127786. doi:10.1016/j.biortech.2022.127786 35970498

[28] Wang F, Fang Y, Wang L, Xiang H, Chen G, Chang X, Liu D, He X, Zhong R. 2022. Effects of residual monensin in livestock manure on nitrogen transformation and microbial community during “crop straw feeding-substrate fermentation-mushroom cultivation” recycling system. Waste Manag 149:333–344. doi:10.1016/j.wasman.2022.06.015 35780758

[29] Zhan Y, Zhang Z, Ma T, Zhang X, Wang R, Liu Y, Sun B, Xu T, Ding G, Wei Y, Li J. 2021. Phosphorus excess changes rock phosphate solubilization level and bacterial community mediating phosphorus fractions mobilization during composting. Bioresour Technol 337:125433. doi:10.1016/j.biortech.2021.125433 34171708

[30] Zhang Q, Zhu T, Xiao Q, An N. 2022 The addition of biochar and hyper-thermal inoculum can regulate the fate of heavy metals resistant bacterial communities during the livestock manure composting. Fermentation 8:207. doi:10.3390/fermentation8050207

[31] Xiao X, Zhong R. 2022. An intelligent integrated system for hyper-thermophilic fermentation of livestock and poultry manure. CN115322017B, issued

[32] Riley D, Barber SA. 1969. Bicarbonate accumulation and ph changes at the soybean (Glycine max (L.) Merr.) Root‐soil interface. Soil Science Soc of Amer J 33:905–908. doi:10.2136/sssaj1969.03615995003300060031x

[33] Adeloju SB, Bond AM, Briggs MH. 1984. Critical evaluation of some wet digestion methods for the stripping voltammetric determination of selenium in biological materials. Anal Chem 56:2397–2401. doi:10.1021/ac00277a031 6517329

[34] Olsen SR, Cole CV, Watanabe FS. 1954. Estimation of available P in soils by extraction with sodium bicarbonate. U.S. Department of Agriculture

[35] Brookes PC, Landman A, Pruden G, Jenkinson DS. 1985. Chloroform fumigation and the release of soil nitrogen: a rapid direct extraction method to measure microbial biomass nitrogen in soil. Soil Biology and Biochemistry 17:837–842. doi:10.1016/0038-0717(85)90144-0

[36] Sparks DL, Sparks DL. 1996. Methods of soil analysis, p 870–874. In Bigham JM (ed), Chemical methods. Soil Science Society of America.

[37] Zhao C, Hu J, Li Q, Fang Y, Liu D, Liu Z, Zhong R. 2022. Transfer of nitrogen and phosphorus from cattle manure to soil and oats under simulative cattle manure deposition. Front Microbiol 13:916610. doi:10.3389/fmicb.2022.916610 35774448 PMC9238326

[38] Tabatabai MA. 1994. Soil enzymes, p 775–883. In Weaver RW, Angle JS, Bottomley PS (ed), Methods of soil analysis: microbiological and bio-chemical properties. Soil Science Society of America.

[39] Eeckhout W, De Paepe M. 1994. Total phosphorus, phytate-phosphorus and phytase activity in plant feedstuffs. Ani Feed Sci Technol 47:19–29. doi:10.1016/0377-8401(94)90156-2

[40] Bolyen E, Rideout JR, Dillon MR. 2018. QIIME 2: reproducible, interactive, scalable, and extensible microbiome data science. Peer J Preprints 6:e27295v2. doi: 10.7287/peerj.preprints.27295v2 10.1038/s41587-019-0209-9 PMC7015180 31341288

[41] Martin M. 2011. Cutadapt removes adapter sequences from high-throughput sequencing reads. EMBnet j 17:10. doi:10.14806/ej.17.1.200

[42] Callahan BJ, McMurdie PJ, Rosen MJ, Han AW, Johnson AJA, Holmes SP. 2016. DADA2: high-resolution sample inference from Illumina amplicon data. Nat Methods 13:581–583. doi:10.1038/nmeth.3869 27214047 PMC4927377

[43] Zelena E, Dunn WB, Broadhurst D, Francis-McIntyre S, Carroll KM, Begley P, O’Hagan S, Knowles JD, Halsall A, HUSERMET Consortium, Wilson ID, Kell DB. 2009. Development of a robust and repeatable UPLC-MS method for the long-term metabolomic study of human serum. Anal Chem 81:1357–1364. doi:10.1021/ac8019366 19170513

[44] Soffer N, Zaneveld J, Vega Thurber R. 2015. Phage-bacteria network analysis and its implication for the understanding of coral disease. Environ Microbiol 17:1203–1218. doi:10.1111/1462-2920.12553 25039472

[45] Ju F, Xia Y, Guo F, Wang Z, Zhang T. 2014. Taxonomic relatedness shapes bacterial assembly in activated sludge of globally distributed wastewater treatment plants. Environ Microbiol 16:2421–2432. doi:10.1111/1462-2920.12355 24329969

[46] Ma B, Wang H, Dsouza M, Lou J, He Y, Dai Z, Brookes PC, Xu J, Gilbert JA. 2016. Geographic patterns of co-occurrence network topological features for soil microbiota at continental scale in eastern China. ISME J 10:1891–1901. doi:10.1038/ismej.2015.261 26771927 PMC5029158

[47] Brown MB. 1975. 400: a method for combining non-independent, one-sided tests of significance. Biometrics 31:987. doi:10.2307/2529826

[48] Hu H-W, Wang J-T, Li J, Shi X-Z, Ma Y-B, Chen D, He J-Z. 2017. Long-term nickel contamination increases the occurrence of antibiotic resistance genes in agricultural soils. Environ Sci Technol 51:790–800. doi:10.1021/acs.est.6b03383 27977160

[49] Wu Y, Si W, Yan S, Wu L, Zhao W, Zhang J, Zhang F, Fan J. 2023. Water consumption, soil nitrate-nitrogen residue and fruit yield of drip-irrigated greenhouse tomato under various irrigation levels and fertilization practices. Agric Water Manag 277:108092. doi:10.1016/j.agwat.2022.108092

[50] Chang D, Song Y, Liang H, Liu R, Cai C, Lv S, Liao Y, Nie J, Duan T, Cao W. 2024. Planting Chinese milk vetch with phosphate-solubilizing bacteria inoculation enhances phosphorus turnover by altering the structure of the phoD-harboring bacteria community. Eur J Soil Biol 123:103678. doi:10.1016/j.ejsobi.2024.103678

[51] Seitz VA, McGivern BB, Borton MA, Chaparro JM, Schipanski ME, Prenni JE, Wrighton KC. 2024. Cover crop root exudates impact soil microbiome functional trajectories in agricultural soils. Microbiome 12:183. doi:10.1186/s40168-024-01886-x 39342284 PMC11439266

[52] Campdelacreu Rocabruna P, Domene X, Matteazzi A, Figl U, Fundneider A, Fernández-Martínez M, Venir E, Robatscher P, Preece C, Peñuelas J, Peratoner G. 2024. Effect of organic fertilisation on soil phosphatase activity, phosphorus availability and forage yield in mountain permanent meadows. Agric Ecosyst Environ 368:109006. doi:10.1016/j.agee.2024.109006

[53] Bi B, Wang K, Zhang H, Wang Y, Fei H, Pan R, Han F. 2021. Plants use rhizosphere metabolites to regulate soil microbial diversity. Land Degrad Dev 32:5267–5280. doi:10.1002/ldr.4107

[54] Hu X, Gu H, Liu J, Wei D, Zhu P, Cui X, Zhou B, Chen X, Jin J, Liu X, Wang G. 2023. Metagenomic strategies uncover the soil bioavailable phosphorus improved by organic fertilization in Mollisols. Agric Ecosyst Environ 349:108462. doi:10.1016/j.agee.2023.108462

[55] Rodríguez-Berbel N, Ortega R, Lucas-Borja ME, Solé-Benet A, Miralles I. 2020. Long-term effects of two organic amendments on bacterial communities of calcareous mediterranean soils degraded by mining. J Environ Manage 271:110920. doi:10.1016/j.jenvman.2020.110920 32579515

[56] Wei X, Hu Y, Cai G, Yao H, Ye J, Sun Q, Veresoglou SD, Li Y, Zhu Z, Guggenberger G, Chen X, Su Y, Li Y, Wu J, Ge T. 2021. Organic phosphorus availability shapes the diversity of phoD-harboring bacteria in agricultural soil. Soil Biol Biochem 161:108364. doi:10.1016/j.soilbio.2021.108364

[57] Dang P, Li C, Lu C, Zhang M, Huang T, Wan C, Wang H, Chen Y, Qin X, Liao Y, Siddique KHM. 2022. Effect of fertilizer management on the soil bacterial community in agroecosystems across the globe. Agric Ecosyst Environ 326:107795. doi:10.1016/j.agee.2021.107795

[58] Ren J, Liu X, Yang W, Yang X, Li W, Xia Q, Li J, Gao Z, Yang Z. 2021. Rhizosphere soil properties, microbial community, and enzyme activities: short-term responses to partial substitution of chemical fertilizer with organic manure. J Environ Manage 299:113650. doi:10.1016/j.jenvman.2021.113650 34481370

[59] Du T, Hu Q, He H, Mao W, Yang Z, Chen H, Sun L, Zhai M. 2023. Long-term organic fertilizer and biofertilizer application strengthens the associations between soil quality index, network complexity, and walnut yield. Eur J Soil Biol 116:103492. doi:10.1016/j.ejsobi.2023.103492

[60] Gu S, Hu Q, Cheng Y, Bai L, Liu Z, Xiao W, Gong Z, Wu Y, Feng K, Deng Y, Tan L. 2019. Application of organic fertilizer improves microbial community diversity and alters microbial network structure in tea (Camellia sinensis) plantation soils. Soil Tillage Research 195:104356. doi:10.1016/j.still.2019.104356

[61] Garrido-Sanz D, Čaušević S, Vacheron J, Heiman CM, Sentchilo V, van der Meer JR, Keel C. 2023. Changes in structure and assembly of a species-rich soil natural community with contrasting nutrient availability upon establishment of a plant-beneficial Pseudomonas in the wheat rhizosphere. Microbiome 11:214. doi:10.1186/s40168-023-01660-5 37770950 PMC10540321

[62] Li H-Z, Peng J, Yang K, Zhang Y, Chen Q-L, Zhu Y-G, Cui L. 2024. Single-cell exploration of active phosphate-solubilizing bacteria across diverse soil matrices for sustainable phosphorus management. Nat Food 5:673–683. doi:10.1038/s43016-024-01024-8 39103543

[63] Liu J, Ma Q, Hui X, Ran J, Ma Q, Wang X, Wang Z. 2020. Long-term high-P fertilizer input decreased the total bacterial diversity but not phoD-harboring bacteria in wheat rhizosphere soil with available-P deficiency. Soil Biol Biochem 149:107918. doi:10.1016/j.soilbio.2020.107918

[64] Liu L, Gao Z, Yang Y, Gao Y, Mahmood M, Jiao H, Wang Z, Liu J. 2023. Long-term high-P fertilizer input shifts soil P cycle genes and microorganism communities in dryland wheat production systems. Agric Ecosyst Environ 342:108226. doi:10.1016/j.agee.2022.108226

[65] Chatterjee D, Nayak AK, Mishra A, Swain CK, Kumar U, Bhaduri D, Panneerselvam P, Lal B, Gautam P, Pathak H. 2021. Effect of long-term organic fertilization in flooded rice soil on phosphorus transformation and phosphate solubilizing microorganisms. J Soil Sci Plant Nutr 21:1368–1381. doi:10.1007/s42729-021-00446-8

[66] Zhang Y, Gao W, Ma L, Luan H, Tang J, Li R, Li M, Huang S, Wang L. 2023. Long-term partial substitution of chemical fertilizer by organic amendments influences soil microbial functional diversity of phosphorus cycling and improves phosphorus availability in greenhouse vegetable production. Agric Ecosyst Environ 341:108193. doi:10.1016/j.agee.2022.108193

[67] Liu X, Zhang Y, Wang Z, Chen Z. 2024. The contribution of organic and chemical fertilizers on the pools and availability of phosphorus in agricultural soils based on a meta-analysis. Eur J Agron 156:127144. doi:10.1016/j.eja.2024.127144

[68] Ran X, Li S, Tejaswi Uppuluri NS, Deng Y, Su Y, Huang G, Dong R, Müller J, Wei Q, Guo J, Oechsner H. 2025. Phosphorus transformation and bioavailability in livestock manure through aerobic composting and anaerobic digestion. Chem Eng J 505:159285. doi:10.1016/j.cej.2025.159285

[69] Dai Z, Liu G, Chen H, Chen C, Wang J, Ai S, Wei D, Li D, Ma B, Tang C, Brookes PC, Xu J. 2020. Long-term nutrient inputs shift soil microbial functional profiles of phosphorus cycling in diverse agroecosystems. ISME J 14:757–770. doi:10.1038/s41396-019-0567-9 31827246 PMC7031380

[70] Li H-P, Han Q-Q, Liu Q-M, Gan Y-N, Rensing C, Rivera WL, Zhao Q, Zhang J-L. 2023. Roles of phosphate-solubilizing bacteria in mediating soil legacy phosphorus availability. Microbiol Res 272:127375. doi:10.1016/j.micres.2023.127375 37058784

[71] Lopes CM, Silva AMM, Estrada-Bonilla GA, Ferraz-Almeida R, Vieira JLV, Otto R, Vitti GC, Cardoso EJBN. 2021. Improving the fertilizer value of sugarcane wastes through phosphate rock amendment and phosphate-solubilizing bacteria inoculation. J Clean Prod 298:126821. doi:10.1016/j.jclepro.2021.126821

[72] Székely AJ, Sipos R, Berta B, Vajna B, Hajdú C, Márialigeti K. 2009. DGGE and T-RFLP analysis of bacterial succession during mushroom compost production and sequence-aided T-RFLP profile of mature compost. Microb Ecol 57:522–533. doi:10.1007/s00248-008-9424-5 18654815

[73] Semenov MV, Krasnov GS, Semenov VM, Ksenofontova N, Zinyakova NB, van Bruggen AHC. 2021. Does fresh farmyard manure introduce surviving microbes into soil or activate soil-borne microbiota? J Environ Manage 294:113018. doi:10.1016/j.jenvman.2021.113018 34144322

[74] Elhaissoufi W, Ghoulam C, Barakat A, Zeroual Y, Bargaz A. 2022. Phosphate bacterial solubilization: a key rhizosphere driving force enabling higher P use efficiency and crop productivity. J Adv Res 38:13–28. doi:10.1016/j.jare.2021.08.014 35572398 PMC9091742

[75] Gusakov AV. 2011. Alternatives to Trichoderma reesei in biofuel production. Trends Biotechnol 29:419–425. doi:10.1016/j.tibtech.2011.04.004 21612834

[76] Grum-Grzhimaylo AA, Falkoski DL, van den Heuvel J, Valero-Jiménez CA, Min B, Choi I-G, Lipzen A, Daum CG, Aanen DK, Tsang A, Henrissat B, Bilanenko EN, de Vries RP, van Kan JAL, Grigoriev IV, Debets AJM. 2018. The obligate alkalophilic soda-lake fungus Sodiomyces alkalinus has shifted to a protein diet. Mol Ecol 27:4808–4819. doi:10.1111/mec.14912 30368956

[77] Luo J, Liao G, Banerjee S, Gu S, Liang J, Guo X, Zhao H, Liang Y, Li T. 2023. Long-term organic fertilization promotes the resilience of soil multifunctionality driven by bacterial communities. Soil Biol Biochem 177:108922. doi:10.1016/j.soilbio.2022.108922

[78] Mao L, Yin B, Ye Z, Kang J, Sun R, Wu Z, Ge J, Ping W. 2024. Plant growth-promoting microorganisms drive K strategists through deterministic processes to alleviate biological stress caused by Fusarium oxysporum. Microbiol Res 289:127911. doi:10.1016/j.micres.2024.127911 39303412

[79] Mogollón JM, Bouwman AF, Beusen AHW, Lassaletta L, van Grinsven HJM, Westhoek H. 2021. More efficient phosphorus use can avoid cropland expansion. Nat Food 2:509–518. doi:10.1038/s43016-021-00303-y 37117673

[80] Xu M, Wang Y, Nie C, Song G, Xin S, Lu Y, Bai Y, Zhang Y, Wang L. 2023. Identifying the critical phosphorus balance for optimizing phosphorus input and regulating soil phosphorus effectiveness in a typical winter wheat–summer maize rotation system in North China. J Integrat Agri 22:3769–3782. doi:10.1016/j.jia.2023.05.030

[81] Li S, Kang J, Wu Z, Sun Y, Tu X, Guo Y, Mao L, Yang Y, Yao W, Ge J. 2025. Mechanisms of phosphorus conversion in chicken manure and straw composting systems regulated by flax-retting wastewater and a combination of flax-retting wastewater and biochar. Chem Eng J 507:160773. doi:10.1016/j.cej.2025.160773

[82] Blundell R, Schmidt JE, Igwe A, Cheung AL, Vannette RL, Gaudin ACM, Casteel CL. 2020. Organic management promotes natural pest control through altered plant resistance to insects. Nat Plants 6:483–491. doi:10.1038/s41477-020-0656-9 32415295

[83] Fan K, Delgado-Baquerizo M, Guo X, Wang D, Zhu Y-G, Chu H. 2021. Biodiversity of key-stone phylotypes determines crop production in a 4-decade fertilization experiment. ISME J 15:550–561. doi:10.1038/s41396-020-00796-8 33028975 PMC8027226

[84] Crowther TW, Rappuoli R, Corinaldesi C, Danovaro R, Donohue TJ, Huisman J, Stein LY, Timmis JK, Timmis K, Anderson MZ, et al.. 2024. Scientists’ call to action: microbes, planetary health, and the sustainable development goals. Cell 187:5195–5216. doi:10.1016/j.cell.2024.07.051 39303686

[85] Zhao Z-B, He J-Z, Geisen S, Han L-L, Wang J-T, Shen J-P, Wei W-X, Fang Y-T, Li P-P, Zhang L-M. 2019. Protist communities are more sensitive to nitrogen fertilization than other microorganisms in diverse agricultural soils. Microbiome 7:33. doi:10.1186/s40168-019-0647-0 30813951 PMC6393985

