# Supplementary material for: Innovative manure via hyper-thermophilic fermentation coupled with heat-resistant phosphate-solubilizing Bacillus inoculation promotes phosphorus transformation by assembling keystone taxa in the oat rhizosphere
Source: Appl Environ Microbiol. 2025 Dec 29;92(1):e01208-25. doi: 10.1128/aem.01208-25 (PMC12838353; doi:10.1128/aem.01208-25)
Supplement: Supplemental material — Tables S1 to S13; Fig. S1 to S16. [file aem.01208-25-s0001.docx]

**Supplemental tables and figures**

**Innovative manure via hyper-thermophilic fermentation coupled with heat-resistant phosphate-solubilizing Bacillus inoculation promotes phosphorus transformation by assembling keystone taxa in the oat rhizosphere**

Chengzhen Zhao^a^, Xiao Chang^a,b^, Lili Fan^a^, Linshu Jiang^a^, Rongzhen Zhong^a,^*

^a^ *Jilin Province Feed Processing and Ruminant Precision Breeding Cross regional Cooperation Technology Innovation Center, Jilin Provincial Laboratory of Grassland Farming, Northeast Institute of Geography and Agroecology, Chinese Academy of Sciences, Changchun 130102, China*

^b^ *University of Chinese Academy of Sciences, Beijing, China*

Corresponding author: Rongzhen Zhong

Address: 4888 Shengbei Street, Changchun 130102, Jilin province, China.

E-mail: zhongrongzhen@iga.ac.cn

Tel: 86-13620790036; 86-18943173671

**Supplemental tables**

Table S1 The physicochemical characteristics of three organic fertilizers. Different letters in the same row mean significant difference at *p < 0.05* among organic fertilizers (Duncan’s test).

| Parameters | Different organic fertilizers | | |  | |  | |  | |
| --- | --- | --- | --- | --- | --- | --- | --- | --- | --- |
|  | Composted manure | | Fermented manure | | Raw manure | | SEM | | *p*-value |
| pH | | 8.43a | 8.21b | | 7.68c | | 0.077 | | <.001 |
| EC (mS/cm) | | 4.17a | 3.94b | | 2.23b | | 0.213 | | <.001 |
| TP (g/kg) | | 6.62b | 7.29a | | 5.95c | | 0.135 | | <.001 |
| AP (g/kg) | | 1.44c | 1.59b | | 1.75a | | 36.075 | | <.001 |
| MBP (mg/kg) | | 72.72b | 140.72a | | 40.41c | | 10.772 | | <.001 |
| ACP (mg PNP/g.24h) | | 49.57a | 23.96b | | 13.17c | | 3.876 | | <.001 |
| NEP (mg PNP/g.24h) | | 53.53a | 13.07b | | 8.99b | | 5.121 | | <.001 |
| ALP (mg PNP/g.24h) | | 252.59a | 166.75b | | 84.46c | | 17.358 | | <.001 |
| Phytase (μg PNP/g.24h) | | 66.69b | 175.99a | | 32.64c | | 15.163 | | <.001 |

Table S2 The additive amount of raw soil and fertilizer among fertilization treatments within each soil type.

| Fertilization treatments | Additive | Moisture  (%) | Wet matter addition (g/pot) | Dry matter addition (g/pot) |
| --- | --- | --- | --- | --- |
| Black soil, |  |  |  |  |
| No manure addition  (CK) | Raw black soil | 16.0 | 5000 | 4200 |
|  | No fertilizer | / | 0 | 0 |
| Composted manure addition (CM) | Raw black soil | 16.0 | 5000 | 4200 |
|  | Composted manure | 65.2 | 575 | 200 |
| Fermented manure addition (FM) | Raw black soil | 16.0 | 5000 | 4200 |
|  | Fermented manure | 60.0 | 500 | 200 |
| Raw manure addition  (RM) | Raw black soil | 16.0 | 5000 | 4200 |
|  | Raw manure | 84.0 | 1250 | 200 |
| Meadow soil, |  |  |  |  |
| No manure addition  (CK) | Raw black soil | 6.7 | 4500 | 4200 |
|  | No fertilizer | / | 0 | 0 |
| Composted manure addition (CM) | Raw black soil | 6.7 | 4500 | 4200 |
|  | Composted manure | 65.2 | 575 | 200 |
| Fermented manure addition (FM) | Raw black soil | 6.7 | 4500 | 4200 |
|  | Fermented manure | 60.0 | 500 | 200 |
| Raw manure addition  (RM) | Raw black soil | 6.7 | 4500 | 4200 |
|  | Raw manure | 84.0 | 1250 | 200 |

Table S3 Information of primers of bacteria and fungi used in this study

|  | Bacteria |  | Fungi |  |
| --- | --- | --- | --- | --- |
| Primers | 338F | 338F | ITS5 | ITS2 |
| Primer sequence  (5’ - 3’) | ACTCCTACGG  GAGGCAGCA | GGACTACHVG  GGTWTCTAAT | GGAAGTAAAAG  TCGTAACAAGG | GCTGCGTTCT  TCATCGATGC |
| Target gene | 16S | | ITS | |
| Target subfragment | V3-V4 region | | ITS region | |
| Subfragment length | 430 | | 350 | |
| PCR reaction condition | An initial denaturation at 98℃ for 5 min, followed by 25 cycles consisting of denaturation at 98 °C for 30 s, annealing at 53°C for 30 s, and extension at 72°C for 45 s, with a final extension of 5 min at 72°C. | | An initial denaturation at 98°C for 5 min, followed by 30 cycles consisting of denaturation at 98°C for 30 s, annealing at 55°C for 30 s, and extension at 72°C for 45 s, with a final extension of 5 min at 72°C. | |

Table S4 The data evaluation of sample sequencing by 16S rRNA

| Samples | Input | Filtered | Denoised | Merged | Non-chimeric | ASV numbers | Q30rate (%) |
| --- | --- | --- | --- | --- | --- | --- | --- |
| Black soil, |  |  |  |  |  |  |  |
| CK1 | 137262 | 124175 | 117047 | 97109 | 86284 | 83343 | 91.29 |
| CK2 | 141334 | 129007 | 121671 | 101617 | 90263 | 87336 | 91.3 |
| CK3 | 138346 | 126521 | 119767 | 101492 | 90864 | 88113 | 91.48 |
| CK4 | 134779 | 121865 | 115340 | 98129 | 88386 | 85978 | 91.16 |
| CK5 | 135729 | 122051 | 115253 | 99186 | 90163 | 87707 | 90.68 |
| CK6 | 129160 | 116097 | 108635 | 88897 | 78907 | 76191 | 90.77 |
| CM1 | 142999 | 128990 | 122447 | 96625 | 79801 | 76902 | 91.24 |
| CM2 | 141967 | 124928 | 118632 | 95231 | 78972 | 76716 | 90.88 |
| CM3 | 139978 | 123413 | 117280 | 93680 | 76505 | 73982 | 90.9 |
| CM4 | 138483 | 124455 | 118277 | 95883 | 81402 | 79032 | 91.47 |
| CM5 | 138466 | 125388 | 118615 | 93054 | 75597 | 72702 | 91.61 |
| CM6 | 130718 | 118649 | 112852 | 87940 | 70335 | 67632 | 91.22 |
| FM1 | 136923 | 125392 | 118436 | 95632 | 80360 | 77189 | 91.32 |
| FM2 | 130612 | 120698 | 114574 | 93226 | 76984 | 73823 | 91.81 |
| FM3 | 144202 | 132852 | 124933 | 99709 | 83797 | 80465 | 91.57 |
| FM4 | 143558 | 131136 | 124847 | 102538 | 87292 | 84375 | 90.99 |
| FM5 | 131942 | 121090 | 113740 | 90526 | 76161 | 72818 | 91.46 |
| FM6 | 139825 | 128921 | 121798 | 97759 | 79853 | 76194 | 91.7 |
| RM1 | 130306 | 119657 | 112600 | 88388 | 74413 | 71279 | 91.57 |
| RM2 | 143674 | 131835 | 124753 | 101334 | 85882 | 82994 | 91.38 |
| RM3 | 140559 | 129009 | 122218 | 98335 | 83303 | 80492 | 91.62 |
| RM4 | 140121 | 128305 | 121984 | 100537 | 85668 | 83141 | 91.63 |
| RM5 | 130287 | 120663 | 114959 | 95567 | 83212 | 80942 | 91.95 |
| RM6 | 142841 | 129918 | 124103 | 103880 | 89524 | 87220 | 91.37 |
| Meadow soil, |  |  |  |  |  |  |  |
| CK1 | 143352 | 133033 | 125478 | 105630 | 94993 | 92518 | 91.92 |
| CK2 | 136822 | 127230 | 120637 | 105268 | 97250 | 95272 | 91.87 |
| CK3 | 135573 | 124489 | 117708 | 98685 | 89286 | 86999 | 91.38 |
| CK4 | 141725 | 129765 | 122390 | 101918 | 90947 | 88561 | 91.19 |
| CK5 | 132295 | 121844 | 114726 | 99350 | 91742 | 89742 | 91.54 |
| CK6 | 126300 | 116362 | 109531 | 96383 | 92013 | 90708 | 91.59 |
| CM1 | 137110 | 123749 | 117743 | 97561 | 86531 | 84714 | 91.6 |
| CM2 | 143744 | 128615 | 123072 | 103272 | 87359 | 85559 | 90.69 |
| CM3 | 130562 | 118188 | 112232 | 90363 | 78140 | 76307 | 91.72 |
| CM4 | 142040 | 127618 | 121803 | 100905 | 85780 | 83882 | 91.31 |
| CM5 | 134090 | 121468 | 115430 | 96775 | 86604 | 85037 | 91.44 |
| CM6 | 144629 | 128756 | 123601 | 107182 | 96411 | 95086 | 90.35 |
| FM1 | 142243 | 130065 | 123518 | 105249 | 86991 | 84698 | 91.08 |
| FM2 | 131663 | 121778 | 114963 | 92969 | 74241 | 71095 | 91.8 |
| FM3 | 135697 | 124506 | 118287 | 98185 | 79914 | 77263 | 91.3 |
| FM4 | 140119 | 128968 | 122134 | 102463 | 84513 | 81756 | 91.4 |
| FM5 | 129982 | 118613 | 112522 | 93296 | 74042 | 71666 | 91.08 |
| FM6 | 130335 | 120700 | 114498 | 97835 | 88435 | 86721 | 91.39 |
| RM1 | 134636 | 124039 | 117055 | 95168 | 84752 | 82615 | 91.69 |
| RM2 | 130135 | 118736 | 112675 | 93349 | 83720 | 81936 | 91.14 |
| RM3 | 142388 | 129713 | 123212 | 99383 | 87946 | 85802 | 91.1 |
| RM4 | 142126 | 129495 | 122699 | 101098 | 91031 | 88974 | 91.44 |
| RM5 | 134243 | 122408 | 116596 | 97643 | 85434 | 83504 | 91.74 |
| RM6 | 140928 | 129465 | 123214 | 103937 | 94059 | 92021 | 91.68 |

Table S5 The data evaluation of sample sequencing by ITS

| Samples | Input | Filtered | Denoised | Merged | Non-chimeric | ASV numbers | Q30rate (%) |
| --- | --- | --- | --- | --- | --- | --- | --- |
| Black soil, |  |  |  |  |  |  |  |
| CK1 | 82370 | 67274 | 66536 | 65218 | 60695 | 60695 | 90.61 |
| CK2 | 81034 | 69311 | 68781 | 67924 | 60125 | 60124 | 90.89 |
| CK3 | 83687 | 61845 | 61314 | 60317 | 53837 | 53837 | 89.57 |
| CK4 | 82963 | 63112 | 62470 | 61410 | 57359 | 57358 | 89.87 |
| CK5 | 87410 | 71172 | 70415 | 69633 | 63360 | 63359 | 90.27 |
| CK6 | 87081 | 61694 | 61066 | 59860 | 53836 | 53836 | 88.88 |
| CM1 | 83569 | 67541 | 67055 | 66478 | 61052 | 61051 | 89.36 |
| CM2 | 83331 | 67152 | 66841 | 65969 | 61054 | 61054 | 89.69 |
| CM3 | 84587 | 67107 | 66645 | 65905 | 59720 | 59720 | 89.13 |
| CM4 | 87370 | 71328 | 70901 | 70045 | 64424 | 64423 | 89.69 |
| CM5 | 81755 | 67367 | 66895 | 66063 | 61425 | 61425 | 89.92 |
| CM6 | 85941 | 70758 | 70406 | 69580 | 63953 | 63953 | 89.72 |
| FM1 | 83946 | 72525 | 72073 | 71174 | 67209 | 67209 | 89.89 |
| FM2 | 81711 | 71158 | 70590 | 68681 | 65674 | 65674 | 90.25 |
| FM3 | 87655 | 72908 | 72502 | 70478 | 67515 | 67514 | 89.16 |
| FM4 | 80207 | 69075 | 68637 | 67938 | 64608 | 64607 | 89.96 |
| FM5 | 81529 | 71195 | 70792 | 69813 | 66034 | 66034 | 90.4 |
| FM6 | 83384 | 70895 | 70424 | 69519 | 65774 | 65773 | 90.14 |
| RM1 | 80357 | 71010 | 70520 | 70051 | 65436 | 65434 | 90.47 |
| RM2 | 87978 | 71096 | 70653 | 70276 | 67756 | 67756 | 88.94 |
| RM3 | 85941 | 75978 | 75620 | 75264 | 71227 | 71226 | 90.57 |
| RM4 | 79953 | 62793 | 62416 | 62047 | 59409 | 59409 | 88.5 |
| RM5 | 84946 | 67781 | 67359 | 66930 | 63324 | 63324 | 88.7 |
| RM6 | 88235 | 61137 | 60769 | 60140 | 57991 | 57991 | 85.86 |
| Meadow soil, |  |  |  |  |  |  |  |
| CK1 | 80264 | 70094 | 69215 | 68272 | 60351 | 60349 | 91.3 |
| CK2 | 83873 | 71331 | 70426 | 68897 | 65506 | 65506 | 90.62 |
| CK3 | 80169 | 68724 | 68097 | 67005 | 61583 | 61583 | 90.62 |
| CK4 | 86854 | 75463 | 74564 | 73124 | 67661 | 67660 | 90.88 |
| CK5 | 82946 | 70498 | 69536 | 68375 | 63995 | 63995 | 90.5 |
| CK6 | 80909 | 67732 | 66727 | 65090 | 60814 | 60814 | 90.5 |
| CM1 | 86832 | 65577 | 65129 | 64156 | 60161 | 60161 | 88.83 |
| CM2 | 80027 | 61591 | 61153 | 59967 | 56163 | 56162 | 88.75 |
| CM3 | 79946 | 62330 | 61853 | 61226 | 56969 | 56969 | 89.39 |
| CM4 | 84449 | 63495 | 63136 | 62493 | 58266 | 58266 | 88.83 |
| CM5 | 84252 | 63315 | 62873 | 61678 | 58418 | 58418 | 88.65 |
| CM6 | 85901 | 66151 | 65767 | 65067 | 61214 | 61214 | 88.93 |
| FM1 | 83952 | 73021 | 72572 | 70084 | 66238 | 66237 | 89.91 |
| FM2 | 81657 | 72481 | 71860 | 70243 | 63037 | 63035 | 90.74 |
| FM3 | 80926 | 69332 | 68464 | 67081 | 63535 | 63535 | 89.87 |
| FM4 | 81995 | 66477 | 65964 | 65035 | 60933 | 60933 | 89.22 |
| FM5 | 82579 | 71636 | 71097 | 69125 | 64226 | 64223 | 90.21 |
| FM6 | 84814 | 74292 | 73747 | 71473 | 66100 | 66100 | 90.22 |
| RM1 | 82062 | 74168 | 73641 | 72864 | 67020 | 67020 | 91.1 |
| RM2 | 86002 | 75028 | 74241 | 73107 | 69944 | 69944 | 90.26 |
| RM3 | 80982 | 72179 | 71717 | 70707 | 66700 | 66699 | 90.36 |
| RM4 | 80439 | 72265 | 71628 | 71074 | 63725 | 63723 | 91.1 |
| RM5 | 87630 | 72369 | 71828 | 70854 | 66656 | 66656 | 89.47 |
| RM6 | 80699 | 71873 | 71463 | 70582 | 65524 | 65524 | 90.25 |

Table S6 The effects of fertilization treatment and soil type on the changes of all plant growth and soil physicochemical properties based on general linear model (GLM)

| Parameters | | | Fertilization |  | Soil type |  | Fertilization × Soil type | |
| --- | --- | --- | --- | --- | --- | --- | --- | --- |
|  |  |  | *F*-value | *P*-value | *F*-value | *P*-value | *F*-value | *P*-value |
| Plant parameters |  |  |  |  |  |  |  |  |
| Aboveground biomass (g/pot) | | | 484.239 | <.001 | 33.005 | <.001 | 4.832 | .006 |
| Aboveground P accumulation (mg/pot) | | | 180.877 | <.001 | 7.386 | .010 | 0.740 | .534 |
| Aboveground P content (g/kg) | | | 116.183 | <.001 | 0.230 | .634 | 7.215 | .001 |
| Belowground biomass (g/pot) | | | 104.283 | <.001 | 39.620 | <.001 | 5.717 | .002 |
| Belowground P accumulation (mg/pot) | | | 264.537 | <.001 | 0.434 | .514 | 3.173 | .034 |
| Belowground P content (g/kg) | | | 1187.692 | <.001 | 509.369 | <.001 | 79.930 | <.001 |
| Soil parameters |  |  |  |  |  |  |  |  |
| pH | | | 44.007 | <.001 | 893.100 | <.001 | 15.123 | <.001 |
| EC (μS/cm) | | | 185.687 | <.001 | 231.708 | <.001 | 45.461 | <.001 |
| Total P content (g/kg) | | | 90.520 | <.001 | 1031.757 | <.001 | 3.639 | .021 |
| Available P content (mg/kg) | | | 477.043 | <.001 | 2.515 | .121 | 2.652 | .062 |
| Microbial biomass P content (mg/kg) | | | 74.704 | <.001 | 2.441 | .126 | 0.649 | .588 |
| Acid phosphatase activity (mg PNP/g.24h) | | | 176.248 | <.001 | 33.069 | <.001 | 3.302 | .030 |
| Neutral phosphatase activity (mg PNP/g.24h) | | | 55.962 | <.001 | 24.262 | <.001 | 5.037 | .005 |
| Alkaline phosphatase activity (mg PNP/g.24h) | | | 266.850 | <.001 | 9.255 | .004 | 4.572 | .008 |
| Phytase activity (μg PNP/g.24h) | | | 44.958 | <.001 | 747.275 | <.001 | 9.059 | <.001 |

Table S7 Basic topological properties of bacterial and fungi occurrence networks with different fertilization treatments based on two soil types. CK: No fertilizer addition; CM composted manure addition; FM: Fermented manure addition; RM: Raw manure addition.

| Topological properties |  |  | All soil type | | | |
| --- | --- | --- | --- | --- | --- | --- |
|  |  |  | CK | CM | FM | RM |
| Average clustering coefficient | | | 0.77 | 0.70 | 0.79 | 0.78 |
| Average path length | | | 1.45 | 1.91 | 1.96 | 1.51 |
| Network diameter | | | 6.00 | 8.00 | 7.00 | 5.00 |
| Network density | | | 0.36 | 0.22 | 0.24 | 0.32 |
| Modularity | | | 0.11 | 0.18 | 0.21 | 0.11 |
| Number of nodes | | | 270 | 210 | 258 | 248 |
| Number of edges | | | 12940 | 4913 | 7994 | 9739 |
| Bacterial nodes | | | 148 | 175 | 138 | 192 |
| Fungal nodes | | | 122 | 35 | 120 | 56 |
| Edges linking bacteria to bacteria | | | 5676 | 3816 | 3127 | 6174 |
| Edges linking bacteria to fungi | | | 5824 | 1009 | 3429 | 3187 |
| Edges linking fungi to fungi | | | 1440 | 88 | 1438 | 378 |

Table S8 The Mantel test between bacteria or fungi and soil physicochemical properties in black soil. TP: total phosphorus; AP: available phosphorus; MBP: microbial biomass phosphorus; ACP: acid phosphatase; NEP: neutral phosphatase; ALP: alkaline phosphatase

| Classification | Soil properties | r | p-value |
| --- | --- | --- | --- |
| Bacteria in black soil | pH | 0.172 | 0.008 |
|  | EC | 0.525 | 0.001 |
|  | TP | 0.406 | 0.001 |
|  | AP | 0.373 | 0.001 |
|  | MBP | 0.328 | 0.001 |
|  | ACP | 0.461 | 0.001 |
|  | NEP | 0.379 | 0.001 |
|  | ALP | 0.463 | 0.001 |
|  | Phytase | 0.173 | 0.005 |
| Fungi in black soil | pH | 0.100 | 0.101 |
|  | EC | 0.574 | 0.001 |
|  | TP | 0.387 | 0.001 |
|  | AP | 0.333 | 0.002 |
|  | MBP | 0.270 | 0.001 |
|  | ACP | 0.539 | 0.001 |
|  | NEP | 0.464 | 0.001 |
|  | ALP | 0.572 | 0.001 |
|  | Phytase | 0.131 | 0.073 |

Table S9 The importance of environmental factors on microorganisms (bacterial phyla and fungal phyla) determined by random forest analysis using increase in the mean square error (InMSE), based on all fertilization treatments (including no manure addition (CK), composted manure addition (CM), fermented manure addition (FM) and raw manure addition (RM)) in a black soil. TP: total phosphorus; AP: available phosphorus; MBP: microbial biomass phosphorus; ACP: acid phosphatase; NEP: neutral phosphatase; ALP: alkaline phosphatase

| Phylum-level microorganism | Soil properties | Increase in MSE (%) | *p-*value |
| --- | --- | --- | --- |
| Actinobacteria | pH | 4.00 | 0.04 |
| Firmicutes | pH | 14.92 | 0.01 |
| Patescibacteria | pH | 6.71 | 0.03 |
| Rozellomycota | pH | 3.81 | 0.04 |
| Proteobacteria | EC | 5.90 | 0.02 |
| Actinobacteria | EC | 10.40 | 0.01 |
| Acidobacteria | EC | 5.62 | 0.04 |
| Planctomycetes | EC | 11.08 | 0.01 |
| Basidiomycota | EC | 7.45 | 0.02 |
| Chytridiomycota | EC | 4.41 | 0.03 |
| Glomeromycota | EC | 4.73 | 0.02 |
| Actinobacteria | TP | 8.64 | 0.01 |
| Acidobacteria | TP | 3.82 | 0.04 |
| Firmicutes | TP | 15.08 | 0.01 |
| Planctomycetes | TP | 8.22 | 0.01 |
| Basidiomycota | TP | 5.56 | 0.03 |
| Proteobacteria | AP | 4.12 | 0.04 |
| Actinobacteria | AP | 6.91 | 0.01 |
| Acidobacteria | AP | 3.87 | 0.04 |
| Firmicutes | AP | 14.85 | 0.01 |
| Planctomycetes | AP | 6.26 | 0.04 |
| Rozellomycota | AP | 4.24 | 0.04 |
| Chytridiomycota | AP | 4.98 | 0.02 |
| Proteobacteria | MBP | 6.41 | 0.02 |
| Actinobacteria | MBP | 9.49 | 0.01 |
| Firmicutes | MBP | 10.88 | 0.01 |
| Planctomycetes | MBP | 6.83 | 0.03 |
| Basidiomycota | MBP | 7.14 | 0.04 |
| Rozellomycota | MBP | 5.18 | 0.01 |
| Proteobacteria | ACP | 5.37 | 0.04 |
| Actinobacteria | ACP | 9.70 | 0.01 |
| Firmicutes | ACP | 6.41 | 0.01 |
| Planctomycetes | ACP | 9.69 | 0.01 |
| Basidiomycota | ACP | 5.43 | 0.02 |
| Mortierellomycota | ACP | 4.40 | 0.03 |
| Proteobacteria | NEP | 5.31 | 0.03 |
| Actinobacteria | NEP | 7.45 | 0.01 |
| Acidobacteria | NEP | 5.26 | 0.04 |
| Bacteroidetes | NEP | 7.43 | 0.03 |
| Planctomycetes | NEP | 8.79 | 0.01 |
| Basidiomycota | NEP | 6.64 | 0.02 |
| Mortierellomycota | NEP | 4.78 | 0.03 |
| Glomeromycota | NEP | 3.55 | 0.01 |
| Proteobacteria | ALP | 5.98 | 0.04 |
| Actinobacteria | ALP | 9.78 | 0.01 |
| Bacteroidetes | ALP | 5.75 | 0.04 |
| Firmicutes | ALP | 5.56 | 0.04 |
| Planctomycetes | ALP | 9.69 | 0.01 |
| Basidiomycota | ALP | 7.63 | 0.02 |
| Glomeromycota | ALP | 3.91 | 0.01 |
| Firmicutes | Phytase | 14.04 | 0.01 |
| Patescibacteria | Phytase | 5.58 | 0.03 |
| Mucoromycota | Phytase | 3.81 | 0.03 |

Table S10 The Mantel test between bacteria or fungi and soil physicochemical properties in meadow soil. TP: total phosphorus; AP: available phosphorus; MBP: microbial biomass phosphorus; ACP: acid phosphatase; NEP: neutral phosphatase; ALP: alkaline phosphatase

| Classification | Soil properties | r | p-value |
| --- | --- | --- | --- |
| Bacteria in meadow soil | pH | 0.416 | 0.001 |
|  | EC | 0.661 | 0.001 |
|  | TP | 0.416 | 0.001 |
|  | AP | 0.616 | 0.001 |
|  | MBP | 0.493 | 0.001 |
|  | ACP | 0.468 | 0.001 |
|  | NEP | 0.397 | 0.001 |
|  | ALP | 0.486 | 0.001 |
|  | Phytase | 0.480 | 0.001 |
| Fungi in meadow soil | pH | 0.371 | 0.001 |
|  | EC | 0.597 | 0.001 |
|  | TP | 0.402 | 0.001 |
|  | AP | 0.586 | 0.001 |
|  | MBP | 0.440 | 0.001 |
|  | ACP | 0.486 | 0.001 |
|  | NEP | 0.422 | 0.001 |
|  | ALP | 0.484 | 0.001 |
|  | Phytase | 0.452 | 0.001 |

Table S11 The importance of environmental factors on microorganisms (bacterial phyla and fungal phyla) determined by random forest analysis using increase in the mean square error (InMSE), based on all fertilization treatments (including no manure addition (CK), composted manure addition (CM), fermented manure addition (FM) and raw manure addition (RM)) in a meadow soil. TP: total phosphorus; AP: available phosphorus; MBP: microbial biomass phosphorus; ACP: acid phosphatase; NEP: neutral phosphatase; ALP: alkaline phosphatase

| Phylum-level microorganism | Soil properties | Increase in MSE (%) | *p-*value |
| --- | --- | --- | --- |
| Acidobacteria | pH | 4.63 | 0.03 |
| Bacteroidetes | pH | 8.77 | 0.02 |
| Firmicutes | pH | 5.35 | 0.02 |
| Gemmatimonadetes | pH | 7.14 | 0.03 |
| Patescibacteria | pH | 5.37 | 0.03 |
| Ascomycota | pH | 4.23 | 0.03 |
| Actinobacteria | EC | 6.78 | 0.02 |
| Acidobacteria | EC | 9.22 | 0.01 |
| Bacteroidetes | EC | 11.15 | 0.01 |
| Firmicutes | EC | 7.81 | 0.01 |
| Patescibacteria | EC | 5.97 | 0.04 |
| Ascomycota | EC | 6.55 | 0.01 |
| Basidiomycota | EC | 9.37 | 0.02 |
| Actinobacteria | TP | 6.09 | 0.02 |
| Acidobacteria | TP | 7.08 | 0.02 |
| Bacteroidetes | TP | 6.40 | 0.02 |
| Firmicutes | TP | 9.10 | 0.01 |
| Basidiomycota | TP | 7.70 | 0.04 |
| Actinobacteria | AP | 6.34 | 0.01 |
| Chloroflexi | AP | 10.05 | 0.01 |
| Firmicutes | AP | 13.65 | 0.01 |
| Gemmatimonadetes | AP | 6.02 | 0.01 |
| Actinobacteria | MBP | 6.17 | 0.02 |
| Chloroflexi | MBP | 8.16 | 0.01 |
| Acidobacteria | MBP | 7.39 | 0.01 |
| Bacteroidetes | MBP | 5.62 | 0.02 |
| Firmicutes | MBP | 13.39 | 0.01 |
| Patescibacteria | MBP | 4.24 | 0.04 |
| Basidiomycota | MBP | 7.68 | 0.02 |
| Actinobacteria | ACP | 8.21 | 0.01 |
| Acidobacteria | ACP | 6.06 | 0.02 |
| Bacteroidetes | ACP | 8.42 | 0.01 |
| Firmicutes | ACP | 9.03 | 0.01 |
| Patescibacteria | ACP | 7.30 | 0.01 |
| Actinobacteria | NEP | 11.19 | 0.01 |
| Bacteroidetes | NEP | 9.26 | 0.02 |
| Firmicutes | NEP | 7.74 | 0.02 |
| Patescibacteria | NEP | 6.25 | 0.03 |
| Actinobacteria | ALP | 8.45 | 0.01 |
| Acidobacteria | ALP | 5.52 | 0.03 |
| Bacteroidetes | ALP | 7.19 | 0.02 |
| Firmicutes | ALP | 10.16 | 0.01 |
| Patescibacteria | ALP | 6.62 | 0.04 |
| Actinobacteria | Phytase | 5.17 | 0.02 |
| Chloroflexi | Phytase | 8.49 | 0.01 |
| Bacteroidetes | Phytase | 6.39 | 0.03 |
| Firmicutes | Phytase | 13.07 | 0.01 |
| Basidiomycota | Phytase | 5.49 | 0.04 |

Table S12 The importance of environmental factors on microorganisms (bacterial genera and fungal genera) determined by random forest analysis using increase in the mean square error (InMSE), based on all fertilization treatments (including no manure addition (CK), composted manure addition (CM), fermented manure addition (FM) and raw manure addition (RM)) in a black soil. TP: total phosphorus; AP: available phosphorus; MBP: microbial biomass phosphorus; ACP: acid phosphatase; NEP: neutral phosphatase; ALP: alkaline phosphatase

| Genus-level microorganism | Soil properties | Increase in MSE (%) | *p-*value |
| --- | --- | --- | --- |
| Thermobifida | pH | 11.50 | 0.01 |
| Bacillus | pH | 8.83 | 0.01 |
| Sodiomyces | pH | 7.34 | 0.02 |
| Thermobifida | EC | 5.58 | 0.01 |
| Fusarium | EC | 6.38 | 0.02 |
| Chaetomium | EC | 7.52 | 0.03 |
| Scutellinia | EC | 6.12 | 0.01 |
| Thermobifida | TP | 8.98 | 0.01 |
| BIrii41 | TP | 7.26 | 0.01 |
| Chryseolinea | TP | 4.94 | 0.02 |
| Bacillus | TP | 7.82 | 0.01 |
| MB_A2_108 | TP | 5.01 | 0.03 |
| Sodiomyces | TP | 9.50 | 0.01 |
| Chaetomium | TP | 7.51 | 0.01 |
| Subgroup_6 | AP | 4.57 | 0.02 |
| Thermobifida | AP | 8.93 | 0.01 |
| BIrii41 | AP | 5.22 | 0.03 |
| Bacillus | AP | 7.94 | 0.02 |
| MB_A2_108 | AP | 4.43 | 0.02 |
| Devosia | AP | 3.38 | 0.04 |
| Sodiomyces | AP | 8.59 | 0.01 |
| Chaetomium | AP | 4.49 | 0.04 |
| Thermobifida | MBP | 10.59 | 0.01 |
| BIrii41 | MBP | 11.45 | 0.01 |
| Bacillus | MBP | 5.15 | 0.01 |
| SBR1031 | MBP | 4.72 | 0.01 |
| MB_A2_108 | MBP | 4.22 | 0.02 |
| Devosia | MBP | 4.65 | 0.03 |
| Sodiomyces | MBP | 4.84 | 0.01 |
| A4b | ACP | 4.05 | 0.01 |
| BIrii41 | ACP | 5.92 | 0.03 |
| MB_A2_108 | ACP | 6.43 | 0.01 |
| Devosia | ACP | 7.39 | 0.01 |
| Sodiomyces | ACP | 8.55 | 0.01 |
| Fusarium | ACP | 6.60 | 0.03 |
| Chaetomium | ACP | 3.91 | 0.04 |
| Pseudogymnoascus | ACP | 4.59 | 0.04 |
| BIrii41 | NEP | 6.70 | 0.01 |
| MB_A2_108 | NEP | 6.41 | 0.01 |
| Devosia | NEP | 5.86 | 0.01 |
| Fusarium | NEP | 6.74 | 0.03 |
| Chaetomium | NEP | 4.43 | 0.02 |
| BIrii41 | ALP | 7.17 | 0.02 |
| MB_A2_108 | ALP | 5.24 | 0.02 |
| Devosia | ALP | 6.55 | 0.02 |
| Fusarium | ALP | 6.29 | 0.03 |
| Chaetomium | ALP | 6.25 | 0.03 |
| Thermobifida | Phytase | 8.13 | 0.01 |
| Bacillus | Phytase | 8.00 | 0.01 |
| Sodiomyces | Phytase | 8.23 | 0.01 |

Table S13 The importance of environmental factors on microorganisms (bacterial genera and fungal genera) determined by random forest analysis using increase in the mean square error (InMSE), based on all fertilization treatments (including no manure addition (CK), composted manure addition (CM), fermented manure addition (FM) and raw manure addition (RM)) in a meadow soil. TP: total phosphorus; AP: available phosphorus; MBP: microbial biomass phosphorus; ACP: acid phosphatase; NEP: neutral phosphatase; ALP: alkaline phosphatase

| Phylum-level microorganism | Soil properties | Increase in MSE (%) | *p-*value |
| --- | --- | --- | --- |
| Thermobifida | pH | 5.41 | 0.03 |
| BIrii41 | pH | 4.32 | 0.02 |
| Chryseolinea | pH | 5.38 | 0.01 |
| Bacillus | pH | 3.54 | 0.03 |
| JG30-KF-CM45 | pH | 10.57 | 0.01 |
| Remersonia | pH | 6.81 | 0.02 |
| Fusarium | pH | 4.24 | 0.04 |
| Mycothermus | pH | 5.06 | 0.02 |
| Thermomyces | pH | 5.52 | 0.01 |
| Zopfiella | pH | 4.79 | 0.04 |
| Pseudogymnoascus | pH | 2.97 | 0.04 |
| A4b | EC | 4.00 | 0.02 |
| Thermobifida | EC | 7.26 | 0.01 |
| BIrii41 | EC | 7.31 | 0.01 |
| Chryseolinea | EC | 6.69 | 0.01 |
| MB-A2-108 | EC | 4.57 | 0.03 |
| Devosia | EC | 5.20 | 0.03 |
| Remersonia | EC | 4.24 | 0.03 |
| Thermomyces | EC | 4.87 | 0.01 |
| Zopfiella | EC | 5.91 | 0.02 |
| Scutellinia | EC | 3.77 | 0.04 |
| Pseudogymnoascus | EC | 4.61 | 0.04 |
| Thermobifida | TP | 7.47 | 0.01 |
| BIrii41 | TP | 7.78 | 0.01 |
| Chryseolinea | TP | 3.89 | 0.04 |
| Devosia | TP | 3.14 | 0.02 |
| Fusarium | TP | 3.54 | 0.04 |
| Thermomyces | TP | 2.85 | 0.04 |
| Thermobifida | AP | 8.01 | 0.01 |
| BIrii41 | AP | 4.71 | 0.02 |
| Chryseolinea | AP | 6.46 | 0.01 |
| Bacillus | AP | 8.66 | 0.01 |
| MB-A2-108 | AP | 5.01 | 0.02 |
| Devosia | AP | 4.19 | 0.02 |
| Sodiomyces | AP | 8.27 | 0.01 |
| Fusarium | AP | 5.46 | 0.01 |
| Zopfiella | AP | 3.09 | 0.03 |
| Cladorrhinum | AP | 3.62 | 0.03 |
| Pseudogymnoascus | AP | 5.71 | 0.03 |
| A4b | MBP | 6.10 | 0.02 |
| Thermobifida | MBP | 13.11 | 0.01 |
| BIrii41 | MBP | 6.41 | 0.01 |
| Chryseolinea | MBP | 5.15 | 0.01 |
| Bacillus | MBP | 5.80 | 0.01 |
| SBR1031 | MBP | 5.03 | 0.03 |
| Sodiomyces | MBP | 4.36 | 0.03 |
| Zopfiella | MBP | 4.12 | 0.02 |
| A4b | ACP | 3.31 | 0.04 |
| BIrii41 | ACP | 5.31 | 0.01 |
| Chryseolinea | ACP | 6.88 | 0.01 |
| Devosia | ACP | 5.56 | 0.02 |
| Fusarium | ACP | 8.71 | 0.01 |
| Thermomyces | ACP | 3.38 | 0.02 |
| Chaetomium | ACP | 4.92 | 0.03 |
| Pseudogymnoascus | ACP | 6.93 | 0.02 |
| A4b | NEP | 3.24 | 0.01 |
| Chryseolinea | NEP | 4.21 | 0.04 |
| MB-A2-108 | NEP | 5.57 | 0.02 |
| Devosia | NEP | 7.79 | 0.03 |
| Chaetomium | NEP | 10.75 | 0.01 |
| Pseudogymnoascus | NEP | 5.86 | 0.02 |
| A4b | ALP | 3.67 | 0.04 |
| BIrii41 | ALP | 5.62 | 0.01 |
| Chryseolinea | ALP | 5.47 | 0.01 |
| Bacillus | ALP | 4.39 | 0.04 |
| MB-A2-108 | ALP | 4.02 | 0.04 |
| Devosia | ALP | 5.91 | 0.04 |
| Fusarium | ALP | 6.53 | 0.02 |
| Chaetomium | ALP | 6.18 | 0.01 |
| Pseudogymnoascus | ALP | 6.17 | 0.02 |
| A4b | Phytase | 6.74 | 0.01 |
| Thermobifida | Phytase | 8.43 | 0.01 |
| BIrii41 | Phytase | 6.20 | 0.01 |
| Chryseolinea | Phytase | 7.11 | 0.01 |
| Bacillus | Phytase | 8.57 | 0.02 |
| SBR1031 | Phytase | 4.63 | 0.03 |
| Remersonia | Phytase | 4.05 | 0.03 |
| Sodiomyces | Phytase | 5.74 | 0.03 |

**Supplemental figures**

**
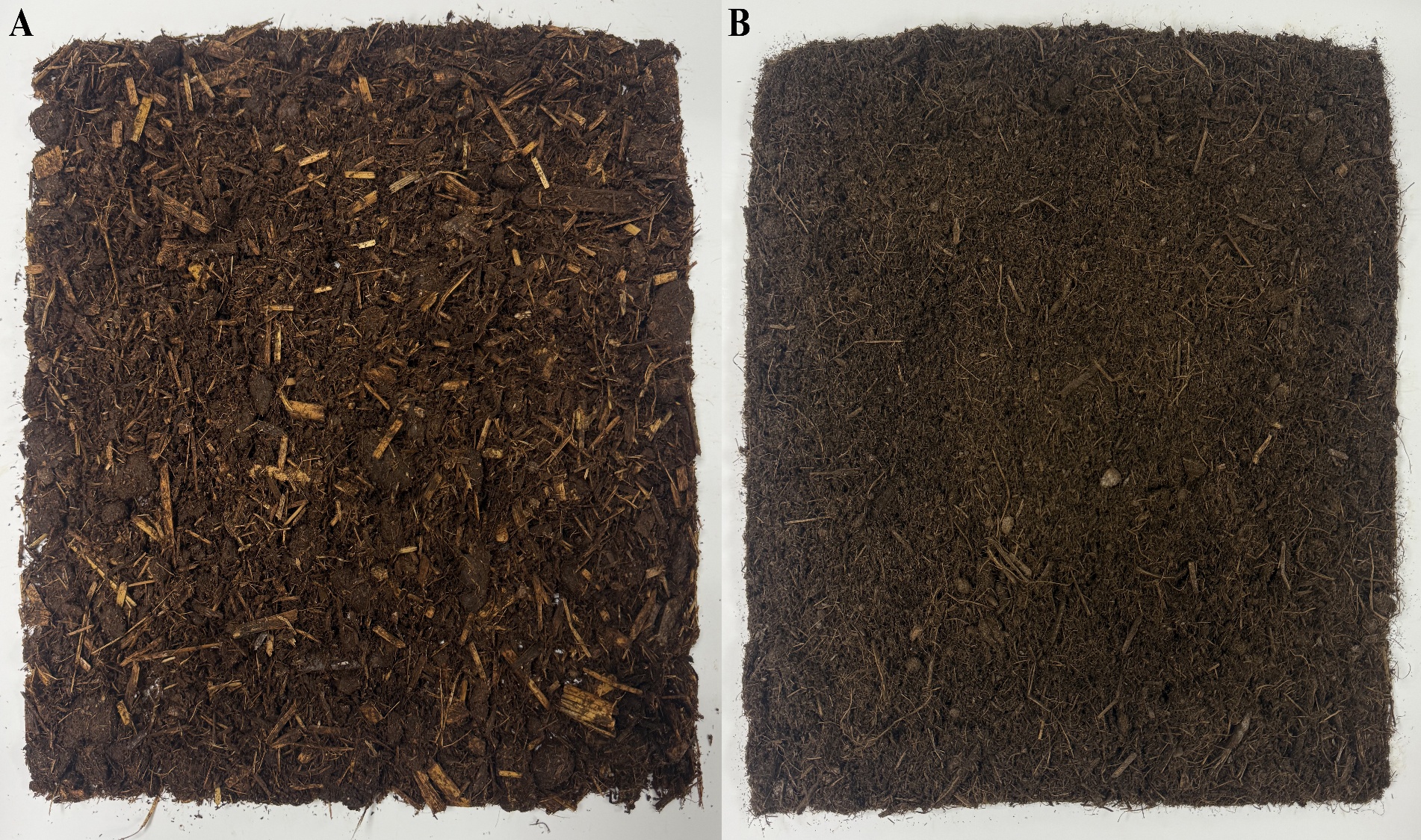
**

Fig. S1 Comparison of the appearance characteristics between conventional aerobic composted manure (A) and hyperthermophilic fermented manure (B).

Fig. S2 Alpha and beta diversity of the bacterial (A, C) and fungal (B, D) community of three organic fertilizers. Alpha diversity metric was estimated by Chao 1 at ASV level. Data represents mean value and upper limit of error bar indicates maximum value within dataset (n=3). The difference in alpha diversity among three organic fertilizers was tested by one-way ANOVA (*P < 0.05*), and only significant differences observed in a comparison group was labeled with letter. Beta diversity was analyzed by nonmetric multidimensional scaling (NMDS) based on Jaccard distance metrics at ASV level.

Fig. S3 Relative abundance of taxonomic composition of organic fertilizer bacterial (A, B) and fungal (C, D) community at both phylum level and genus level.

**
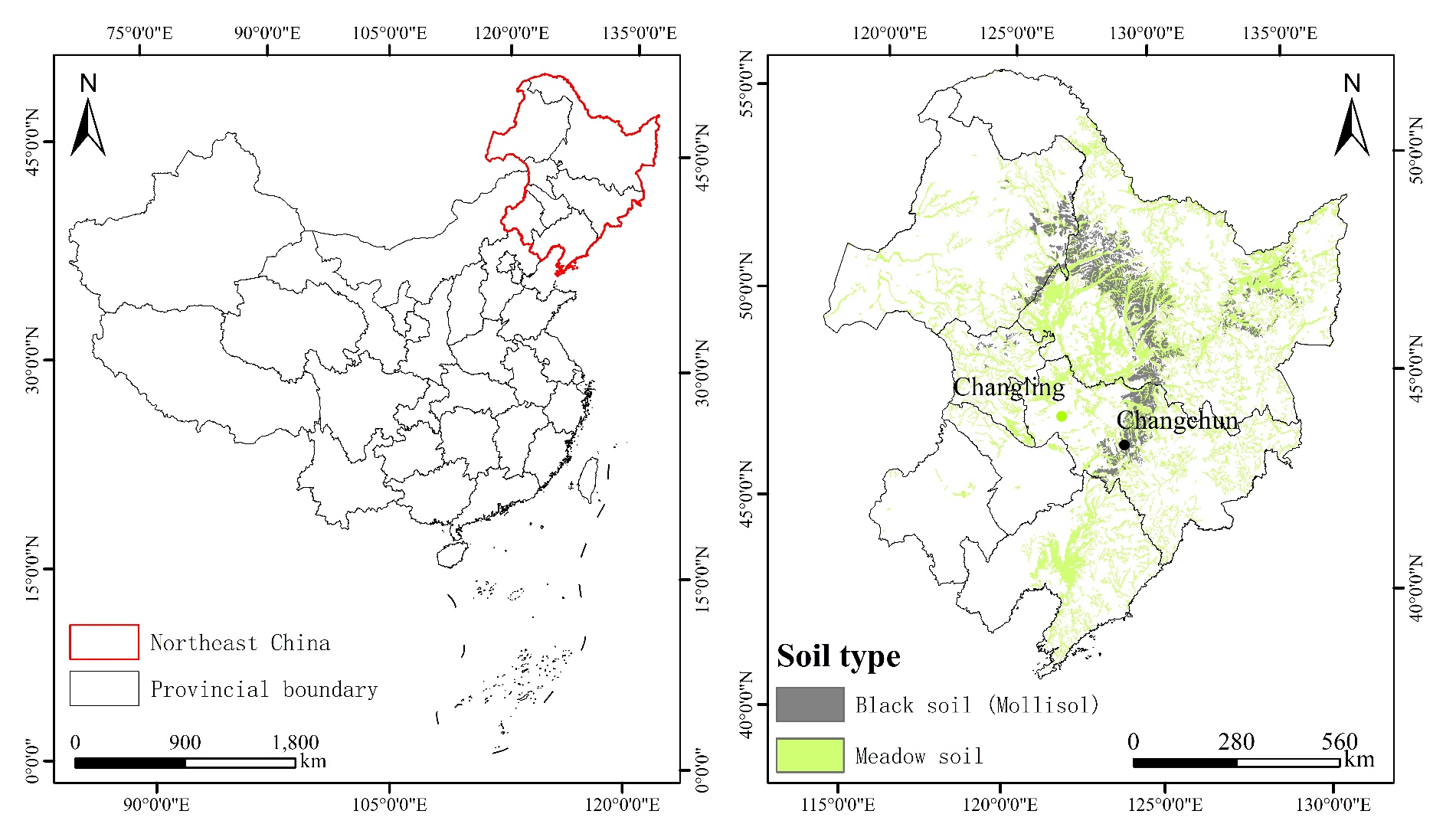
**

Fig. S4 Geographic location of two raw soil collection sites in Northeast China. Two sites located in Changchun and Changling, corresponding to the typical black soil and meadow soil areas, respectively.

**
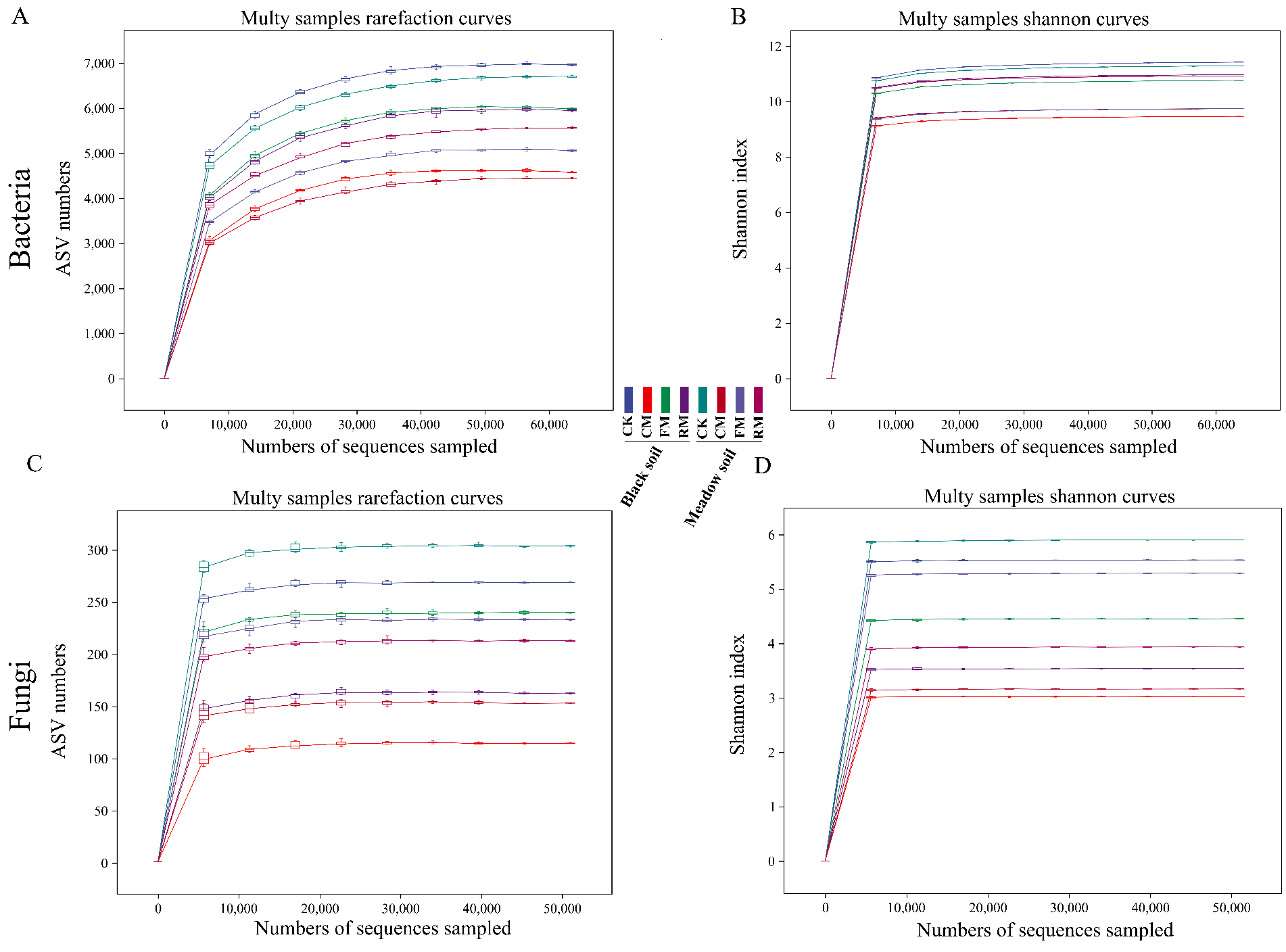
**

Fig. S5 The rarefaction curves and Shannon-Wiener showing the relationship between the sequencing depth and the number of ASVs in each soil sample from black soil or meadow soil for bacteria and fungi, respectively. CK: No fertilizer addition; CM composted manure addition; FM: Fermented manure addition; RM: Raw manure addition.

Fig. S6 Relative abundance of taxonomic composition of soil bacterial (A) and fungal (B) community at genus level among four fertilization treatments within each soil type. CK: No fertilizer addition; CM composted manure addition; FM: Fermented manure addition; RM: Raw manure addition. Four fertilization treatments were applied into black soil and meadow soil, respectively in a pot experiment.

Fig. S7 Relative abundance of the ten most dominant bacteria at phylum level (A-J) in rhizosphere soil among four fertilization treatments within two soil types. The difference in relative abundance of each bacterium among all treatments together was tested by one-way ANOVA (*P < 0.05*), and only significant differences observed in a comparison group was labeled with letter. Data represents mean value and upper limit of error bar indicates maximum value within dataset (n = 6). CK: No fertilizer addition; CM composted manure addition; FM: Fermented manure addition; RM: Raw manure addition. Four fertilization treatments were applied into black soil and meadow soil, respectively in a pot experiment.

Fig. S8 Relative abundance of the ten most dominant bacteria at genus level (A-J) in rhizosphere soil among four fertilization treatments within two soil types. The difference in relative abundance of each bacterium among all treatments together was tested by one-way ANOVA (*P < 0.05*), and only significant differences observed in a comparison group was labeled with letter. Data represents mean value and upper limit of error bar indicates maximum value within dataset (n = 6). CK: No fertilizer addition; CM composted manure addition; FM: Fermented manure addition; RM: Raw manure addition. Four fertilization treatments were applied into black soil and meadow soil, respectively in a pot experiment.

Fig. S9 Relative abundance of the ten most dominant fungal at phylum level (A-J) in rhizosphere soil among four fertilization treatments within two soil types. The difference in relative abundance of each bacterium among all treatments together was tested by one-way ANOVA (*P < 0.05*), and only significant differences observed in a comparison group was labeled with letter. Data represents mean value and upper limit of error bar indicates maximum value within dataset (n = 6). CK: No fertilizer addition; CM composted manure addition; FM: Fermented manure addition; RM: Raw manure addition. Four fertilization treatments were applied into black soil and meadow soil, respectively in a pot experiment.

Fig. S10 Relative abundance of the ten most dominant fungi at genus level (A-J) in rhizosphere soil among four fertilization treatments within two soil types. The difference in relative abundance of each bacterium among all treatments together was tested by one-way ANOVA (*P < 0.05*), and only significant differences observed in a comparison group was labeled with letter. Data represents mean value and upper limit of error bar indicates maximum value within dataset (n = 6). CK: No fertilizer addition; CM composted manure addition; FM: Fermented manure addition; RM: Raw manure addition. Four fertilization treatments were applied into black soil and meadow soil, respectively in a pot experiment.

Fig. S11 Phylum-level composition of the dominant module I and module II in four fertilization treatments rhizosphere networks across all soil type together.

**
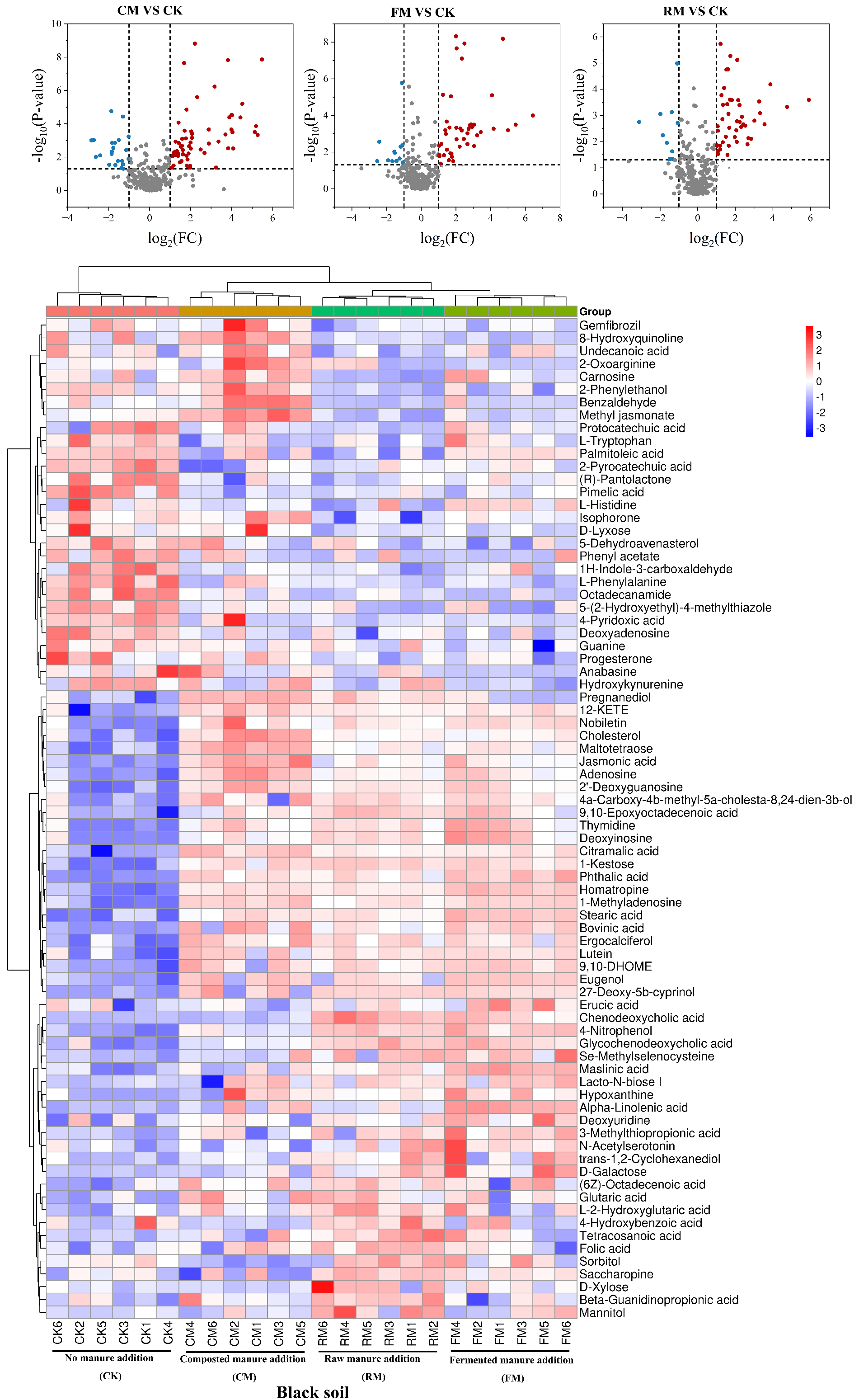
**

Fig. S12 Significantly different metabolites among fertilization treatments (including no manure addition (CK), composted manure addition (CM), fermented manure addition (FM) and raw manure addition (RM)) in black soil. Volcano plots showed the differential metabolites in pairwise comparisons between three fertilization treatments and CK treatment. The upregulated and downregulated metabolites were shown in red and blue, respectively, whereas gray dots represented the metabolites without significance in each group. Heatmap showing expression profiles of each soil sample based on all significant known metabolites identified by ANOVA. Metabolites and soil samples are clustered by hierarchical clustering analysis. Clustered soil samples were classified and displayed in different colored squares, directly above the heatmap, and the corresponding fertilization treatments of colored squares were displayed directly below the heatmap. The color of squares linking metabolites to samples ranges blue to red, indicating metabolite concentration z-score: numbers -3 to 3 on the scale bar indicate the number of standard deviations from the mean.

**
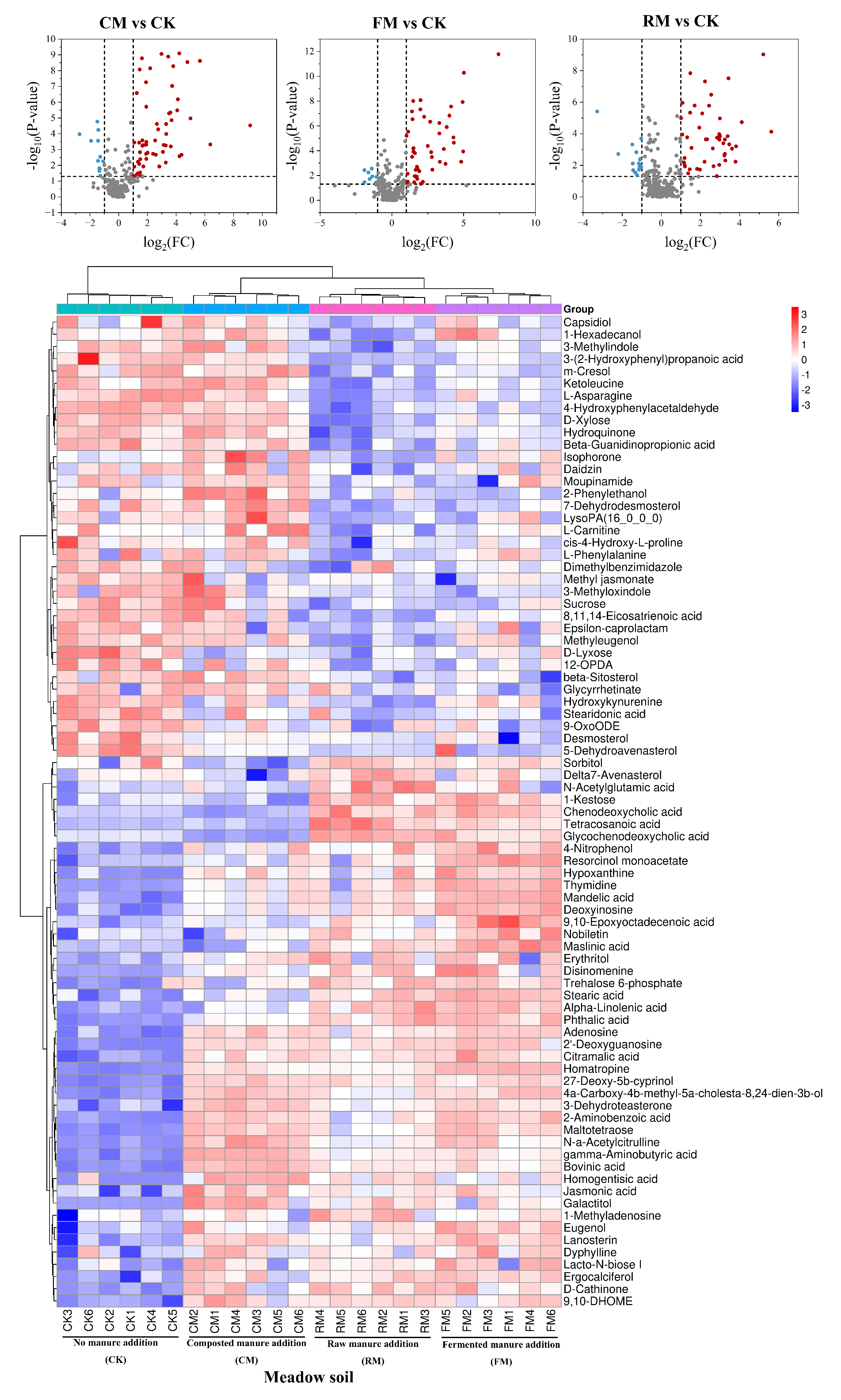
**

Fig S13 Significantly different metabolites among fertilization treatments (including no manure addition (CK), composted manure addition (CM), fermented manure addition (FM) and raw manure addition (RM)) in meadow soil. Volcano plots showed the differential metabolites in pairwise comparisons between three fertilization treatments and CK treatment. The upregulated and downregulated metabolites were shown in red and blue, respectively, whereas gray dots represented the metabolites without significance in each group. Heatmap showing expression profiles of each soil sample based on all significant known metabolites identified by ANOVA. Metabolites and soil samples are clustered by hierarchical clustering analysis. Clustered soil samples were classified and displayed in different colored squares, directly above the heatmap, and the corresponding fertilization treatments of colored squares were displayed directly below the heatmap. The colors of squares linking metabolites to samples ranges blue to red, indicating metabolite concentration z-score: numbers -3 to 3 on the scale bar indicate the number of standard deviations from the mean.

Fig S14 KEGG enrichment analysis of different soil metabolites from pairwise comparisons between three fertilization treatments and CK treatment in black soil. A, C, E showed the up-regulated (red) and down-regulated (blue) pathways between CM treatment and CK treatment, FM treatment and CK treatment, and RM treatment and CK treatment, respectively. B, D, F showed the most significantly 20 pathways enriched by differentially metabolites between CM treatment and CK treatment, FM treatment and CK treatment, and RM treatment and CK treatment, respectively. Red and green colors indicate higher and lower p-value, respectively. The size of the circle represents the number of enriched metabolites. CK: No fertilizer addition; CM composted manure addition; FM: Fermented manure addition; RM: Raw manure addition.

Fig S15 KEGG enrichment analysis of different soil metabolites from pairwise comparisons between three fertilization treatments and CK treatment in Meadow soil. A, C, E showed the upregulated (red) and downregulated (blue) pathways between CM treatment and CK treatment, FM treatment and CK treatment, and RM treatment and CK treatment, respectively. B, D, F showed the most significantly 20 pathways enriched by differentially metabolites between CM treatment and CK treatment, FM treatment and CK treatment, and RM treatment and CK treatment, respectively. Red and green colors indicate higher and lower p-value, respectively. The size of the circle represents the number of enriched metabolites. CK: No fertilizer addition; CM composted manure addition; FM: Fermented manure addition; RM: Raw manure addition.

**
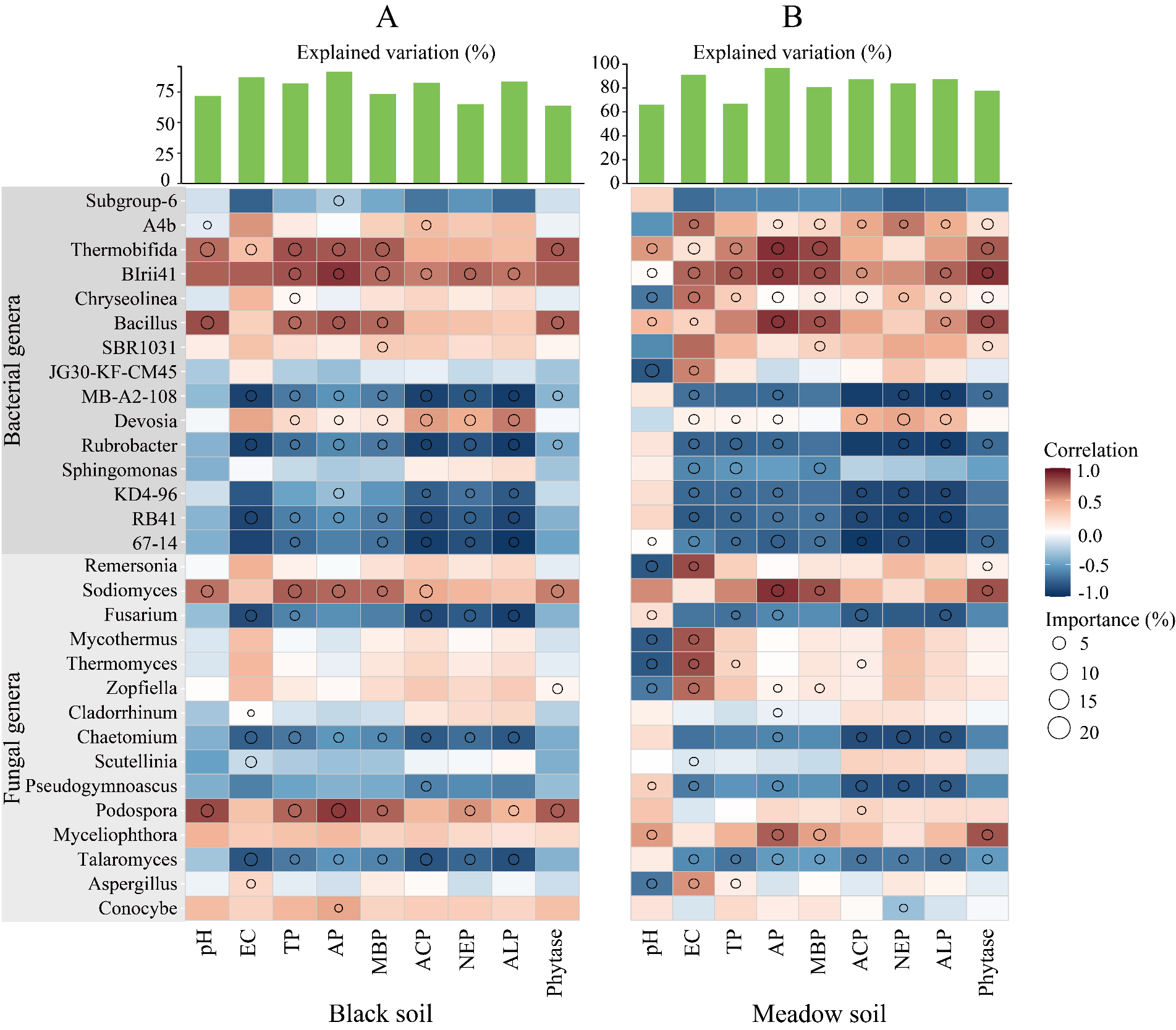
**

Fig. S16 Random Forest analysis of environmental factors and differential microorganisms (bacterial genera and fungal genera) based on all fertilization treatments (including no manure addition (CK), composted manure addition (CM), fermented manure addition (FM) and raw manure addition (RM)) in a black soil (A) and meadow soil (B), respectively. Circle size represents the variable’s importance. Colors represent Spearman’s correlations. The total explanatory power of the differential microorganisms on environmental factors is displayed as a histogram above the heatmap. TP: total phosphorus; AP: available phosphorus; MBP: microbial biomass phosphorus; ACP: acid phosphatase; NEP: neutral phosphatase; ALP: alkaline phosphatase
