# Supplementary figures and images for: Innovative manure via hyper-thermophilic fermentation coupled with heat-resistant phosphate-solubilizing Bacillus inoculation promotes phosphorus transformation by assembling keystone taxa in the oat rhizosphere
Source: Appl Environ Microbiol. 2025 Dec 29;92(1):e01208-25. doi: 10.1128/aem.01208-25 (PMC12838353; doi:10.1128/aem.01208-25)

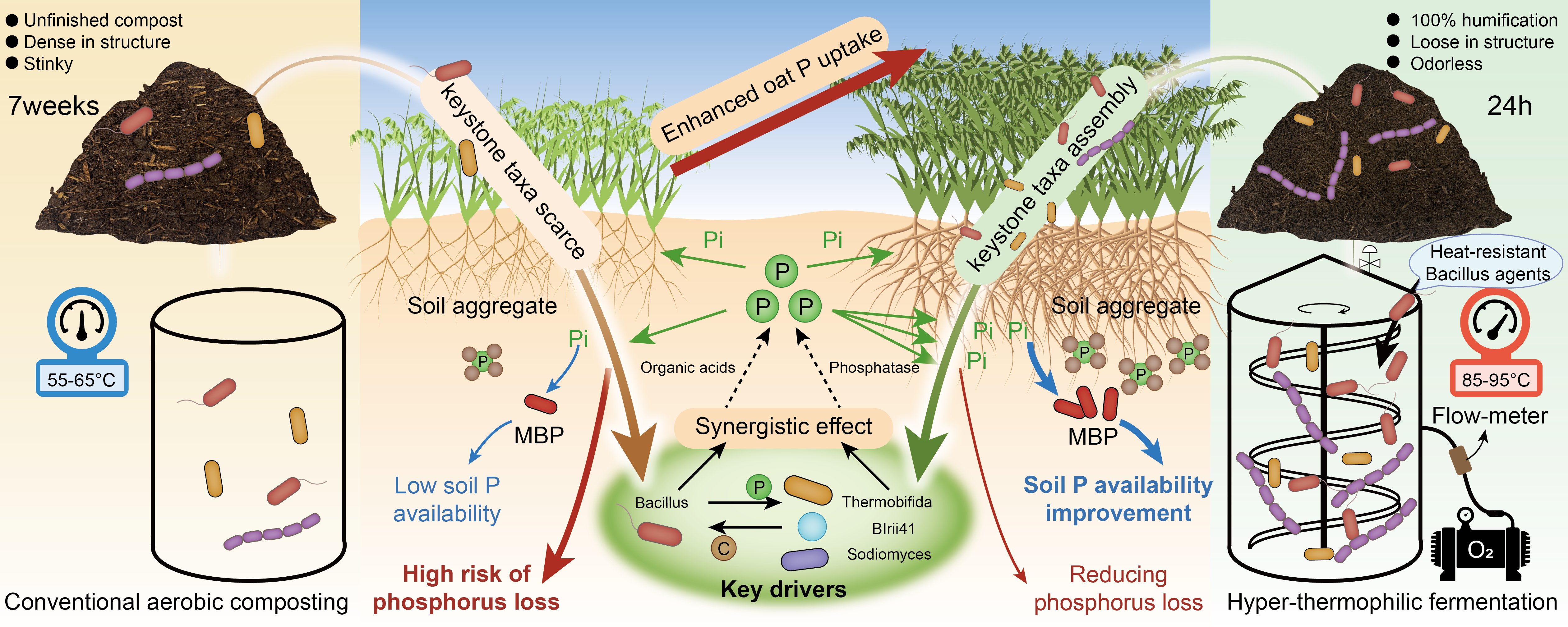

Supplement: Graphical abstract — Visual depiction of the study. [file aem.01208-25-s0002.tif]
